# Photoinduced Ring‐Opening Polymerization of *N*‐Carboxyanhydrides for the Preparation of Cross‐Linked Polypeptide Gels

**DOI:** 10.1002/anie.202521891

**Published:** 2025-12-18

**Authors:** Ana Kočman, David Pahovnik, Ema Žagar, Petra Utroša

**Affiliations:** ^1^ Department of Polymer Chemistry and Technology National Institute of Chemistry Hajdrihova 19 Ljubljana 1000 Slovenia; ^2^ Faculty of Chemistry and Chemical Technology University of Ljubljana Večna pot 113 Ljubljana 1000 Slovenia

**Keywords:** *N*‐carboxyanhydride, Photobase, Photochemistry, Polypeptide gel, Ring‐opening polymerization

## Abstract

Using light to activate chemical reactions is a powerful approach that allows spatiotemporal control over physicochemical transformations, commonly used in the polymerization and solidification of reactive liquids. Here, we employ a photochemical approach for ring‐opening polymerization (ROP) of α‐amino acid *N*‐carboxyanhydride (NCA) monomers to prepare covalently cross‐linked synthetic polypeptide gels using light. We apply 2‐nitrobenzyl carbamate as a photocaged amine (base), where the switch in its activity after illumination is a key factor in controlling ROP. The released dibutylamine, as a basic species, can deprotonate the NCA and thereby initiate polymerization. We investigate the effects of 2‐nitrobenzyl dibutyl carbamate on γ‐benzyl‐l‐glutamate NCA polymerization and apply this approach to prepare covalently cross‐linked polypeptide gels. We demonstrate excellent 2D spatiotemporal control over gelation, further enhanced by the use of an acid inhibitor that prevents overcuring caused by diffusion of active species from the illuminated region. The development of photoinduced polymerization and cross‐linking of NCAs offers significant advantages in polypeptide synthesis and holds great potential for further applications in additive manufacturing.

## Graphical abstract

We employ a photochemical approach for ring‐opening polymerization (ROP) of α‐amino acid *N*‐carboxyanhydride (NCA) monomers to prepare covalently cross‐linked synthetic polypeptide gels using a photobase and light, which offers significant advantages in the synthesis of polypeptides and holds great potential for applications requiring spatial and temporal control, such as additive manufacturing.

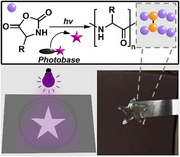

## Introduction

In recent years, photopolymerization has gained significant attention in the field of advanced materials.^[^ ^1^ ^]^ The ability of light to provide both spatial and temporal control makes it an attractive energy source for initiating polymerizations.^[^ ^2^ ^]^ A growing interest in photopolymerizations is attributed to their economic and environmental benefits, including rapid production of materials and lower energy costs, as well as the potential for implementation in additive manufacturing.^[^ ^3^ ^]^ In the latter case, radical polymerizations are typically used to prepare polymer materials with C–C bonds in the backbone, resulting in limited (bio)degradability.^[^ ^4^ ^]^ In contrast, polymers with improved degradability can be prepared by ring‐opening polymerization (ROP) of heterocyclic monomers, as the resulting polymer backbones contain ester, carbonate, or amide bonds that are susceptible to hydrolytic and / or enzymatic degradation.^[^ ^5^ ^]^ ROP of *N*‐carboxyanhydrides (NCAs) provides a simple and convenient route for the preparation of high molar mass polypeptides derived from natural and non‐natural α‐amino acids.^[^ ^6^, ^7^, ^8^ ^]^ This polymerization approach allows the incorporation of functional groups into the polymer structure and makes polypeptides ideal for various biomedical applications due to their biocompatibility, biodegradability, and customizable properties.^[^ ^9^, ^10^, ^11^ ^]^ Synthetic polypeptides can be cross‐linked to enhance their mechanical properties and stability.^[^ ^12^, ^13^ ^]^ Moreover, the use of light in the polypeptide preparation enables the production of cross‐linked polypeptides, which is of great value for use in tissue engineering, where structural accuracy is important.^[^ ^14^ ^]^

The photoinduced ROP of NCAs has not yet been widely explored. Commonly used photoactive molecules for uncaging reactions are carbamate‐based compounds that are decarboxylated under irradiation to release primary or secondary amine.^[^ ^15^, ^16^ ^]^ So far, only primary amines have been released as nucleophilic initiators from 2‐nitrobenzyl carbamate‐based molecules, triggering the ROP of NCAs by the normal amine mechanism (NAM). Heise et al.^[^ ^17^ ^]^ prepared photocaged amines based on 2‐nitrobenzyl carbamates (2,6‐dinitrobenzyl cyclohexyl carbamate and 4,5‐dimetoxy‐2‐nitrobenzyl cyclohexyl carbamate), which released cyclohexylamine upon exposure to light. This approach was further applied to synthesize polypeptides on silicon wafers grafted with carbamate‐photocaged amine,^[^ ^18^ ^]^ as well as hyperbranched polypeptides using *N*_ε_‐(*o*‐nitrobenzyl‐oxycarbonyl)‐l‐lysine NCA inimer molecules,^[^ ^19^, ^20^ ^]^ where released amine successfully initiated the ROP of NCA monomers in all cases, but with limited control over the molar mass and molar mass distribution due to incomplete release of the free amine. Additionally, side reactions occurred as nitrosobenzaldehyde formed imine‐terminated chain ends through reactions with the amine chain‐ends during irradiation period. Sumerlin et al.^[^ ^21^ ^]^ addressed these issues by using 2‐(2‐nitrophenyl) propyloxycarbonyl as a photocage, which improved the release rate of hexylamine, resulting in a more controlled ROP of NCA and elimination of side reactions involving nitrosobenzaldehyde. In these cases, ROP of NCA monomers was successfully initiated by free amines released upon light, however, monomer conversion was below 40% after 2 h. The NAM mechanism generally leads to well‐defined polypeptides, but high molar masses are achieved in a relatively long reaction time and only if no impurities are present in the reaction mixture that could cause uncontrolled chain initiation or termination.^[^ ^8^ ^]^ Fast ROP of NCAs can be achieved with basic catalysts such as tertiary amines, guanidines, sterically hindered secondary amines, etc., which trigger the activated monomer mechanism (AMM) of NCA polymerization.^[^ ^22^, ^23^ ^]^ When secondary amines are used, the NAM and AMM mechanisms often coexist. Nevertheless, AMM usually prevails due to the faster propagation.^[^ ^8^ ^]^ While this results in a loss of control over molar mass and its distribution, it enables the production of high molar mass polypeptides in a much shorter time.^[^ ^23^ ^]^ High molar mass is crucial for the preparation of cross‐linked polypeptides because it ensures sufficient chain length for effective network formation and improved mechanical properties.^[^ ^24^, ^25^ ^]^ To our knowledge, the use of photoinduced ROP of NCAs for the preparation of cross‐linked polypeptides has not been reported previously. We see a great potential in photoinduced polypeptide cross‐linking during polymerization for advancing the field of 3D printing, as only post‐polymerization photocross‐linking has so far been utilized in additive manufacturing of polypeptide materials using light.^[^ ^26^, ^27^, ^28^, ^29^ ^]^

In this work, we demonstrate the preparation of cross‐linked polypeptide gels by photoinduced ROP of NCAs using monofunctional γ‐benzyl‐l‐glutamate (BLG) NCA as monomer and di‐functional l‐homocystine (HCys) NCA as a cross‐linker. We investigated the use of photocaged 2‐nitrobenzyl carbamate bases, which release a free base upon exposure to light that triggers NCA polymerization.

## Results and Discussion

**Figure 1:**
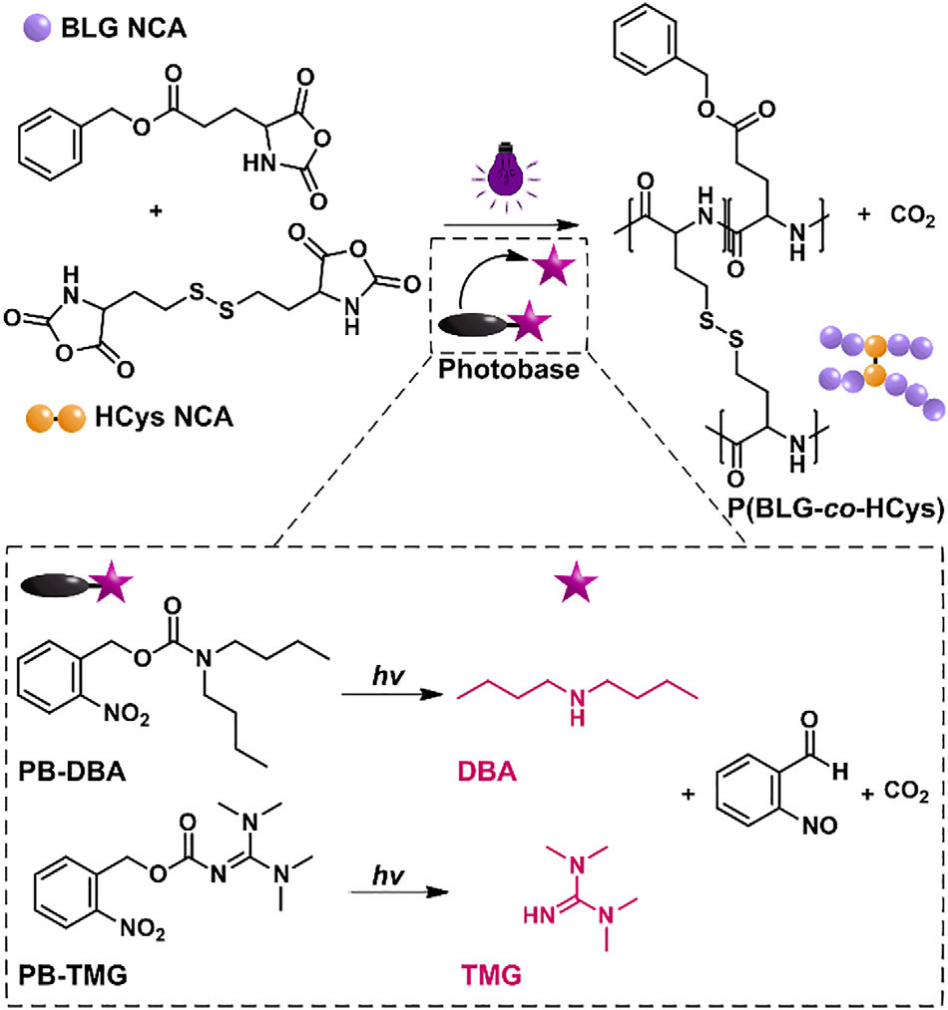
Reaction scheme of the photoinduced ring‐opening copolymerization of BLG NCA and HCys NCA, triggered by the base (DBA or TMG) released from the photobase (PB‐DBA or PB‐TMG) upon irradiation, as depicted in the dashed frame.

To prepare cross‐linked polypeptide gels, we performed photoinduced ring‐opening copolymerization of monofunctional BLG NCA and difunctional cross‐linker HCys NCA (Figure 1). A photoactive catalyst or initiator is required to trigger photoinduced ROP (photo‐ROP). We investigated 2‐nitrobenzyl carbamate‐based photobases which are known to release a caged base (amine) upon irradiation at 365 nm,^[^ ^30^ ^]^ where the investigated NCAs do not absorb light (Figure S10). We investigated two 2‐nitrobenzyl carbamate‐based photobases that release either the stronger base 1,1,3,3‐tetramethylguanidine (TMG) – PB‐TMG, or the weaker base dibutylamine (DBA) – PB‐DBA) (Figure 1), both of which are able to deprotonate NCA monomers at the 3‐N position to initiate polymerization. One of the key advantages of using light to trigger ROP is the ability to control the reaction spatially and temporally i.e., ROP of NCA only starts upon illumination and proceeds exclusively in the areas exposed to light, while no polymerization takes place in the non‐irradiated areas. However, temporal precision can be compromised if premature polymerization takes place due to the high reactivity of NCAs, as they are highly susceptible to nucleophilic attack, usually by impurities or water^[^ ^8^ ^]^ and can also undergo solvent‐induced polymerization in certain solvents, particularly at high monomer concentrations.^[^ ^7^ ^]^ On the other hand, diffusion of active species (base or NCA anion) can deteriorate spatial control. To inhibit unwanted reactions in *N*,*N*‐dimethylformamide (DMF) solutions at high NCA concentrations (1.0 M), we included a small amount of trifluoroacetic acid (TFA, 0.5 mol % relative to NCA), as strong acids such as methanesulfonic acid,^[^ ^31^ ^]^ hydrochloric acid,^[^ ^32^ ^]^ and TFA^[^ ^33^ ^]^ are known to successfully inhibit NCA polymerization. Our polymerization system thus consists of BLG NCA as the monomer, HCys NCA as the difunctional cross‐linker, PB‐TMG or PB‐DBA as the photoactive catalyst, and TFA as the inhibitor, using DMF as the solvent.

**Figure 2:**
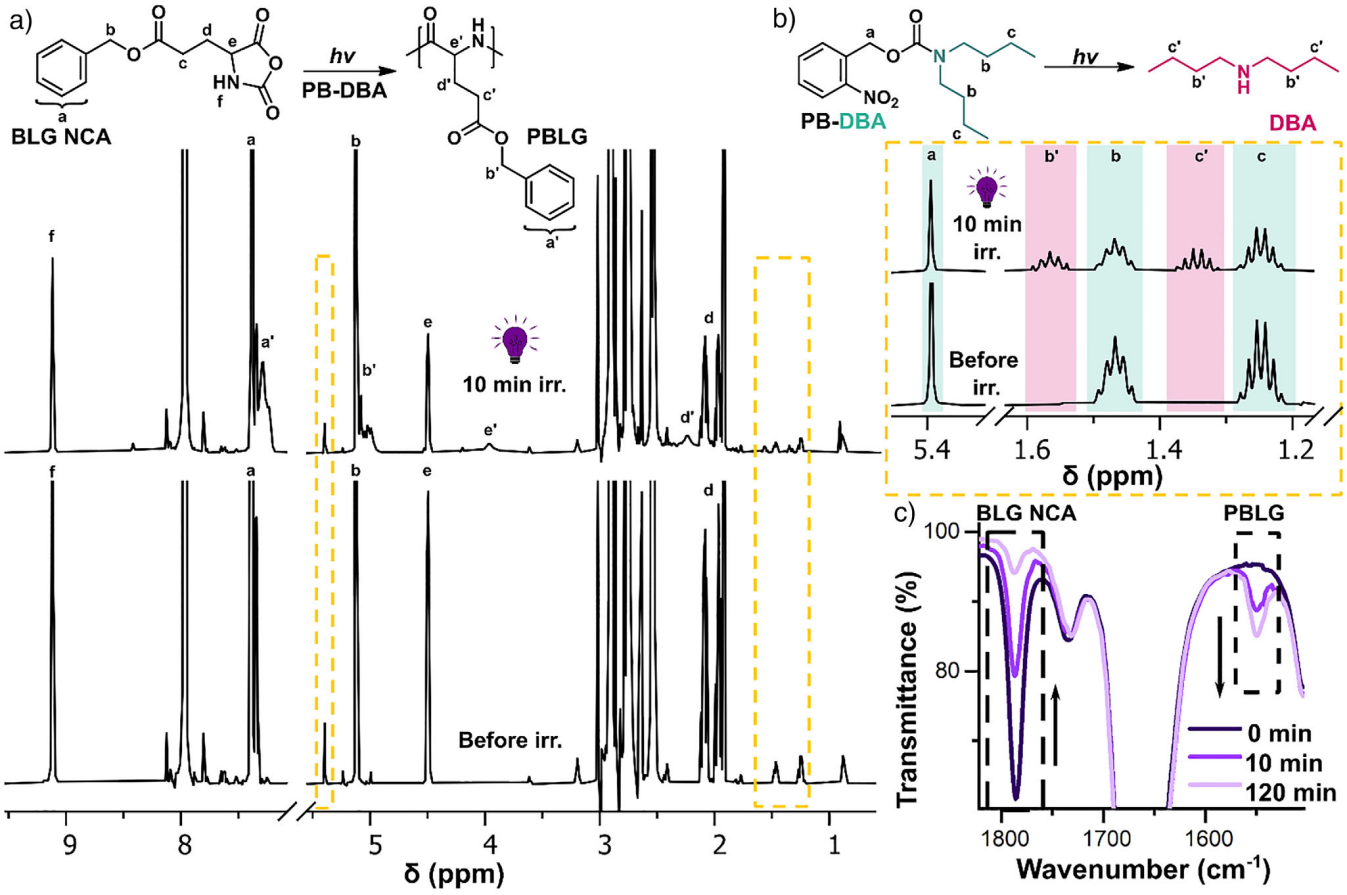
a) ^1^H NMR spectra before and after 10 min of irradiation show the disappearance of BLG NCA and the formation of PBLG. PB‐DBA and DBA signals are marked with dashed yellow rectangles. b) ^1^H NMR spectra of PB‐DBA before and after 10 min of irradiation show the disappearance of PB‐DBA (marked with green rectangles) and the release of DBA (marked with pink rectangles). The dashed yellow rectangles in Figure A indicate the regions of the spectra that are magnified in Figure B. c) Typical FTIR spectra of BLG NCA reaction mixture in DMF at different times (before and after 10 min of irradiation) depicting the disappearance of the BLG NCA carbonyl band at 1787 cm^−1^ and the increase of the polypeptide amide II band at 154 cm^−1^ over time.

Before using the proposed polymerization system for preparation of covalently cross‐linked gels, we first investigated the effects of the selected photobases on the ROP of BLG NCA and characterized the resulting linear polypeptides. It is crucial for the photobase to be inert towards NCA prior to illumination, which PB‐TMG failed to achieve, as polypeptide formed over time even in the absence of light (Figure S11) and was therefore considered unsuitable for further use. Notably, PB‐TMG had been extensively purified and contains no free TMG, as indicated by its ^1^H and ^13^C NMR spectra (Figures S8 and S9), demonstrating that the photocaged base PB‐TMG itself is sufficiently basic to trigger NCA polymerization. Although TMG‐carbamate photobases have shown to be inactive before irradiation in reactions such as thiol‐Michael addition catalysis or olefin metathesis deactivation by base^[^ ^34^, ^35^ ^]^ we hereby highlight the high reactivity of NCA monomers which requires careful selection of the photoactive compound for ROP. On the other hand, PB‐DBA did not trigger the polymerization of BLG NCA in the absence of light within one hour (Figure S11), providing better compatibility with NCA which is essential for the spatial control over the photochemical system. We investigated photochemical reactivity of PB‐DBA under the conditions used for ROP in more detail. Upon irradiation with UV light, hydrogen abstraction and intramolecular rearrangement occur at the excited 2‐nitrobenzyl chromophore, leading to cleavage of the carbamate group and release of free amine/base, CO_2_ and nitrosobenzaldehyde (Figure 1).^[^ ^30^ ^]^ We monitored the ongoing reaction qualitatively by observing a change in solution color from colorless to yellow due to increased absorbance around 385 nm by the nitrosobenzaldehyde formed (Figure S12).^[^ ^36^ ^]^ The degree of photobase decomposition was calculated from ^1^H NMR spectra (Figure 2b) using the integral ratio of the methylene (–C*H*_2_–) signals of PB‐DBA at δ 5.38 ppm at time *t* and at time *t*_0_, relative to the solvent (DMF) peak at δ 7.95 ppm. The conversion of PB‐DBA to DBA was 42% after irradiation with light of 365 nm wavelength for 10 min (corresponding to 54.0 J·cm^−2^). When the light was switched off and the solution was left in the dark, no further photobase decomposition was observed.

**Figure 3:**
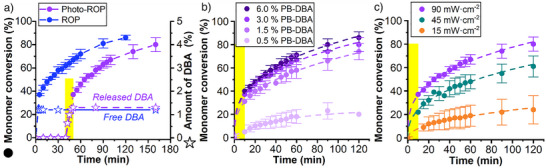
a) Monomer conversion as a function of time (filled circle symbols) for photo‐ROP of BLG NCA with PB‐DBA (3.0%) under 10 min irradiation (365 nm at 90 mW·cm^−2^) and for ROP of BLG NCA with DBA (1.2%) without irradiation. The concentration profile of (released) DBA is shown with empty star symbols. b) Monomer (BLG NCA) conversion as a function of time at different initial PB‐DBA concentrations under 10 min irradiation (365 nm at 90 mW·cm^−2^). c) Monomer (BLG NCA) conversion as a function of time with 3.0% PB‐DBA under 10 min irradiation at 365 nm at different light intensities. The illumination period for all samples is depicted with yellow rectangle.

We further focused on the photo‐ROP of BLG NCA using PB‐DBA as a photocatalyst under irradiation with 365 nm LED light for 10 min. We monitored the polymerization kinetics with FTIR spectroscopy during and after irradiation. As free DBA is released from PB‐DBA during irradiation, we observed a decrease in the height of the BLG NCA carbonyl band at a wavenumber of 1787 cm^−1^ and an increase in the height of the polymer amide band at 1550 cm^−1^ (Figure 2c), which allowed us to calculate the monomer conversion from the linear dependence of the height ratio of monomer to polymer bands. The resulting values for monomer conversion agree well with those obtained by ^1^H NMR (Figure 2a) from the integral ratio of the polymer signal (PBLG at δ 5.11 ppm) and the monomer signals (BLG NCA at δ 5.11 ppm and 4.47 ppm). The results show that PB‐DBA successfully triggered the ROP of NCA during 10 min of irradiation, resulting in 80% monomer conversion after 120 min. No polymerization was observed when the BLG NCA solution was irradiated under the same conditions in the absence of PB‐DBA (Figure S13), confirming the stability of BLG NCA at 365 nm and the necessity of the released DBA as a catalyst. As a control, we performed a “conventional” ROP of BLG NCA, i. e., without irradiation, using 1.2% DBA as a catalyst. The amount of DBA in the conventional ROP matched the amount of DBA released during the photo‐ROP experiment, in which the PB‐DBA conversion after irradiation was 42 %, corresponding to 1.2% released DBA relative to NCA. The reaction kinetics of photo‐ROP proved to be comparable to that triggered by free DBA (Figure 3a). Furthermore, the polypeptides prepared with PB‐DBA and DBA have comparable molar mass averages and molar mass distributions after reaching 100% monomer conversion (*M*_w_ = 45.5 kg·mol^−1^, *Đ* = 1.19 and *M*_w_ = 40.9 kg·mol^−1^, *Đ* = 1.24, respectively), as shown by the SEC/MALS‐RI results (Figure S14), indicating that polymerization with PB‐DBA proceeds without any specific photoinduced side reactions.

Next, we investigated the influence of the initial amount of PB‐DBA on the BLG NCA polymerization kinetics. A higher initial amount of PB‐DBA resulted in faster reaction kinetics (Figure 3b) due to the increased concentration of DBA, which is gradually released during irradiation (Figure S15a). However, the conversion of PB‐DBA to DBA after 10 min irradiation was inversely proportional to the initial PB‐DBA concentration (Figure S15b), possibly due to limited light penetration caused by partial light absorption by the photoproduct or self‐quenching at higher PB‐DBA concentrations.^[^ ^37^ ^]^ While increasing the initial PB‐DBA concentration from 0.5% to 1.5% resulted in a large increase in the absolute amount of DBA released, further increases to 6.0% PB‐DBA had little effect on the release of free DBA. In addition, the final amount of DBA released also depended on the irradiation dose (Figures S16a and S16b), which allowed us to adjust the polymerization kinetics with light intensity. Figure 3c shows that polymerization was fastest at the highest light intensity, as a 10 min irradiation at 90 mW·cm^−2^ (54.0 J·cm^−2^) led to 1.35% DBA released relative to BLG NCA, while at 15 mW·cm^−2^ (9.0 J·cm^−2^) only 0.69% DBA relative to BLG NCA was available to catalyse ROP.

**Figure 4:**
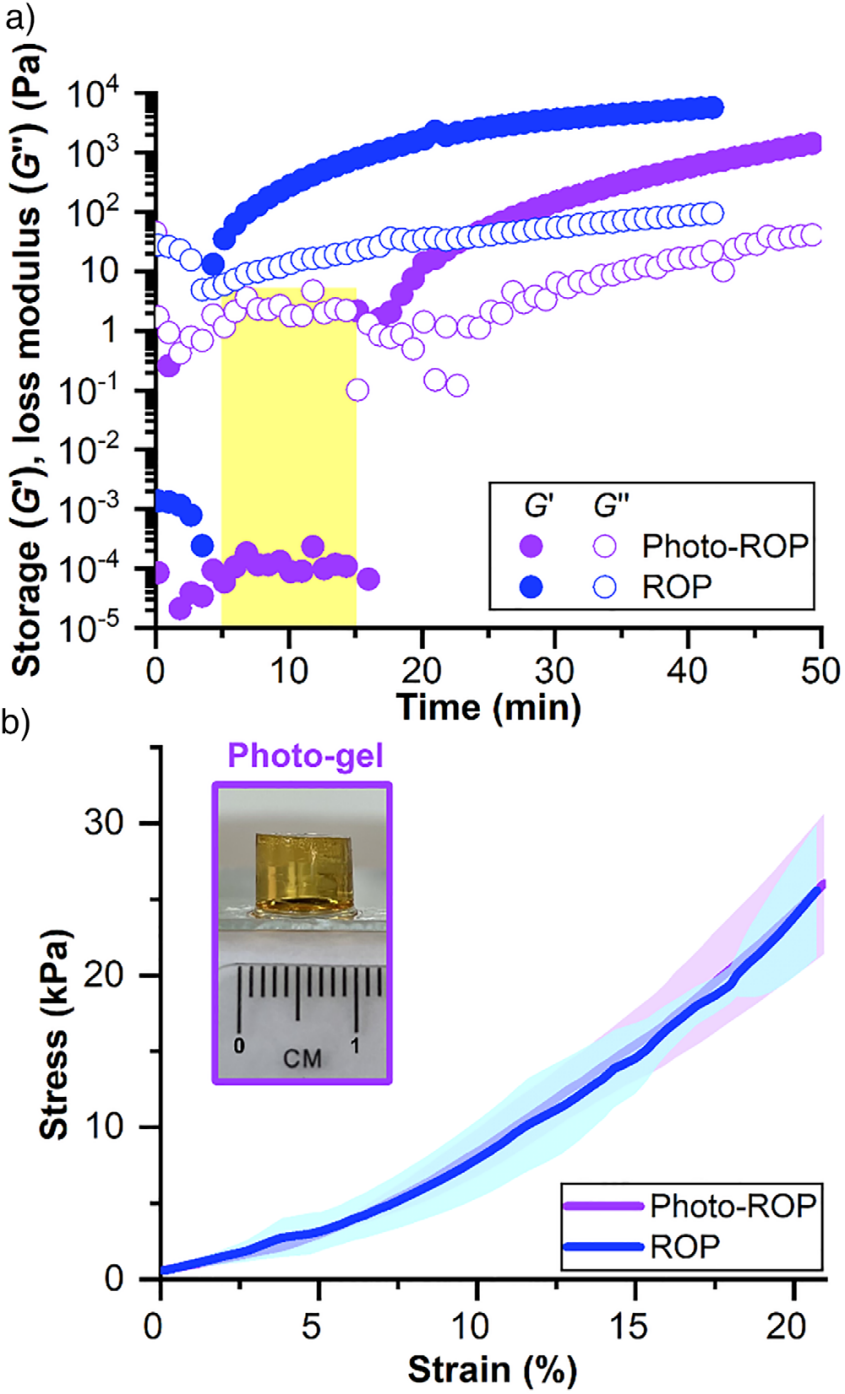
a) Time‐dependent oscillatory rheological test results for the ring‐opening copolymerization of BLG NCA and HCys NCA: Photo‐ROP with PB‐DBA under 10 min irradiation and ROP with DBA without irradiation. b) Compressive stress‐strain curves of P(BLG‐*co*‐HCys) gels triggered by PB‐DBA (photo‐ROP) or DBA (ROP). The curves represent the average of three measurements, with standard deviations indicated. The purple rectangle shows an image of a photogel.

The fast polymerization of NCA by the described photochemical approach is important for the formation of polypeptide gels with uniform network and suitable properties,^[^ ^25^ ^]^ which is essential for advanced manufacturing processes of these materials. Photogels were prepared by photo‐ROP using PB‐DBA as a photocatalyst and monofunctional BLG NCA and difunctional HCys NCA, which is a highly effective cross‐linker due to its stability and lack of decomposition side reactions during ROP with amines.^[^ ^25^ ^]^ Poly(γ‐benzyl‐l‐glutamate‐*co*‐l‐homocystine) (P(BLG‐*co*‐HCys)) formed a photogel after 14 min of reaction time, as shown by UV‐mediated time‐dependent dynamic oscillatory rheological tests, which revealed the onset of crossover of storage modulus (*G*′) over loss modulus (*G*″) (Figure 4a). Temporal control over the gelation of P(BLG‐*co*‐HCys) using PB‐DBA is evident from the gel point occurring 14 min after the start of irradiation, even when the time at which the light source is turned on is delayed by 15 min (Figure S17). The onset of gel formation in photo‐ROP was slower than in conventional ROP, which uses free DBA and formed a P(BLG‐*co*‐HCys) gel within 4 min (Figure 4a), possibly due to the slower reaction kinetics (Figure S18) of photoinduced ROP. However, HCys NCA proved stable under the irradiation conditions used in this work, as ^1^H NMR spectroscopy confirmed that no changes occurred in either irradiated (10 min at 365 nm) or non‐irradiated solutions of HCys NCA after 60 min (Figure S19). Nevertheless, gels prepared with free DBA and PB‐DBA exhibited comparable mechanical properties under compression (Figure 4b). The photogel prepared with PB‐DBA exhibited a compressive modulus of 150 ± 20 kPa, compared to 200 ± 30 kPa for the DBA gel. Since the polymerization kinetics depends on the amount of DBA released, which is directly related to the initial PB‐DBA concentration and the light energy dose used, as with linear polypeptides, these parameters also influenced the gelation point of the cross‐linked polypeptides. Thus, the gel point is reached faster at higher light intensity (25 min at 45 mW·cm^−2^ vs. 50 min at 15 mW·cm^−2^) (Figure S20a) and at higher PB‐DBA loading (11 min at 60 mM PB‐DBA vs. 25 min at 15 mM PB‐DBA) (Figure S20b). The gels exhibited relatively high gel contents (≥ 75%) after removal of the soluble fraction consisting of polypeptide chains that were not covalently incorporated into the network. Together with similar degrees of swelling (4–5 g/g) (Figures S21a and S21b), these results indicate effective network formation in all cases.

**Figure 5:**
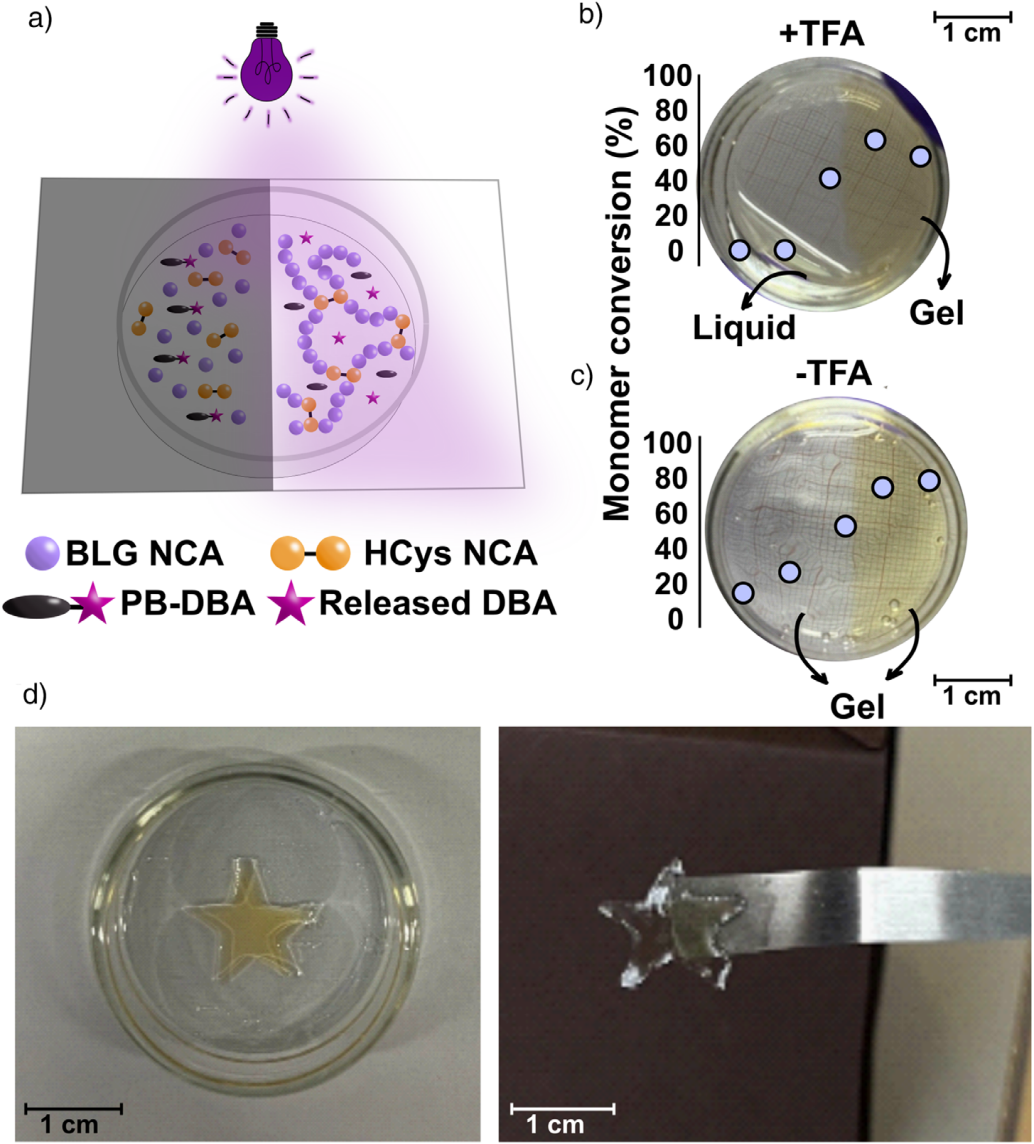
a) Schematic illustration of the photomask experiment to demonstrate spatial control. b) Image of the photomask experiment with TFA (showing gel formation on the irradiated region and liquid on the non‐irradiated region of the reaction mixture), and c) image of the photomask experiment without TFA (showing gel formation in both the irradiated and non‐irradiated regions of the reaction mixture), both after 12 min irradiation (365 nm, 60 mW·cm^−2^). The purple points represent the calculated monomer conversions along the sample. d) Photographs of the gel after the star‐shaped photomask experiment.

In the following, we demonstrated the spatial control of photo‐ROP of NCAs using a photomask experiment. An approximately 0.8 mm thick layer of the reaction mixture (BLG NCA and HCys NCA with 3.0 mol % PB‐DBA and 0.5% TFA) was covered with a photomask that blocks UV light in a predefined pattern, allowing only a certain area of the sample to be illuminated (Figures 5a and S22). The reaction solution was irradiated for 12 min with a light intensity of 60 mW·cm^−2^, corresponding to energy dose of 43.2 J·cm^−2^. After irradiation, only the illuminated part exhibited a yellow color indicative of the 2‐nitrobenzyl photoproducts and, importantly, gel formation was observed only on the illuminated side, while the non‐irradiated side remained liquid (Figure 5b). FTIR measurements performed along the sample from the non‐irradiated to the irradiated side (Figure S23a) confirmed that no polymerization occurred in the non‐illuminated region, with a distinct increase in monomer conversion to 62% at the transition to the illuminated part of the sample (Figures 5b, S23b, and S23c). It is important to note that the ROP of NCA continues until all of the monomer is consumed. Active species formed during and after irradiation such as DBA, NCA anions and –NH_2_ end group bearing species can diffuse from the illuminated region to the non‐illuminated region and trigger polymerization there as well, which would deteriorate spatial control and consequently make it difficult to produce materials with more complex shapes.^[^ ^38^ ^]^ Therefore, it is important to efficiently inhibit the ROP in the non‐illuminated region to maintain spatiotemporal control. The addition of TFA to our reaction mixture proved to be crucial for the inhibition of ROP outside the illuminated region. When no TFA was used, a gel formed in both the irradiated and non‐irradiated regions (Figure 5c). While the NCA in the illuminated region reached 86% conversion without the addition of TFA, polymerization continued gradually in the non‐illuminated region, reaching 20% NCA conversion at the farthest point from the light (Figures 5c, S23b, and S23d). By adding TFA, it was possible to control polymerization in more complex shape as well, as demonstrated with a star‐shaped photomask. In this case, after irradiation, the unreacted liquid surrounding the irradiated pattern was removed, leaving only a star‐shaped gel with well‐resolved features (Figures 5d and S24). These results demonstrate that cross‐linked polypeptide gels can be formed using light and a suitable photobase (PB‐DBA). Temporal and spatial control of the photochemical reaction is possible by optimizing experimental parameters, including the irradiation dose, the initial concentration of PB‐DBA, and the addition of a small amount of a strong acid.

## Conclusion

We expand the photocross‐linking possibilities of synthetic polypeptides via photo‐ROP of NCAs. We have investigated the use of photobases based on 2‐nitrobenzyl carbamates that release the active base (amine) upon irradiation at 365 nm, which triggers ROP of NCAs. An appropriate choice of photobase proved crucial for achieving good control over photo‐ROP. Specifically, PB‐TMG is too basic itself and triggers polymerization of NCAs even in the absence of light, while PB‐DBA serves as an efficient photocaged catalyst that remains inactive in the dark and releases the active catalyst DBA only when the reaction mixture is exposed to light to trigger ROP. Importantly, there was no significant influence of photoproducts on the polypeptides prepared by photo‐ROP compared to those from conventional ROP, as evident from similar polymerization kinetics profiles, polypeptide molar mass characteristics, and mechanical properties of gels. Moreover, the polymerization kinetics and cross‐linking rates can be tuned by the initial photobase concentration as well as light intensity, while the final polypeptide gels exhibit comparable mechanical properties irrespective of these initial parameters. We have achieved good temporal and spatial control over the photo‐ROP, resulting in the preparation of well‐resolved 2D polypeptide gels. This photochemical approach offers significant advantages in the synthesis of polypeptides and holds great potential for applications requiring spatial and temporal control, such as additive manufacturing.

## Supporting Information

The authors have cited additional references within the Supporting Information.^[^ ^39^, ^40^ ^]^

## Conflict of Interests

The authors declare no conflict of interest.

## Supporting information



Supporting Information

## Data Availability

The data that support the findings of this study are available from the corresponding author upon reasonable request.

## References

[1] X. He , L. Zang , Y. Xin , Y. Zou , Appl. Res. 2023, 2, e202300030, 10.1002/appl.202300030.

[2] M. Lang , S. Hirner , F. Wiesbrock , P. Fuchs , Polymers 2022, 14, 2074, 10.3390/polym14102074. 35631956 PMC9145830

[3] N. Guy , O. Giani , S. Blanquer , J. Pinaud , J. J. Robin , Prog. Org. Coat. 2021, 153, 106159, 10.1016/j.porgcoat.2021.106159.

[4] H. Lai , J. Zhang , F. Xing , P. Xiao , Chem. Soc. Rev. 2020, 49, 1867–1886, 10.1039/C9CS00731H. 32101186

[5] G. Becker , F. R. Wurm , Chem. Soc. Rev. 2018, 47, 7739–7782. 30221267 10.1039/c8cs00531a

[6] D. Huesmann , K. Klinker , M. Barz , Polym. Chem. 2017, 8, 957–971, 10.1039/C6PY01817C.

[7] H. R. Kricheldorf , Angew. Chem. Int. Ed. 2006, 45, 5752–5784. 10.1002/anie.200600693 16948174

[8] N. Hadjichristidis , H. Iatrou , M. Pitsikalis , G. Sakellariou , Chem. Rev. 2009, 109, 5528–5578. 19691359 10.1021/cr900049t

[9] Y. Liu , D. Li , J. Ding , X. Chen , Chin. Chem. Lett. 2020, 31, 3001–3014, 10.1016/j.cclet.2020.04.029.

[10] H. Feng , J. Fabrizi , J. Li , C. Mayer , Int. J. Mol. Sci. 2022, 23, 5042, 10.3390/ijms23095042. 35563433 PMC9104059

[11] C. Deng , J. Wu , R. Cheng , F. Meng , H. A. Klok , Z. Zhong , Prog. Polym. Sci. 2014, 39, 330–364.

[12] A. Rasines Mazo , S. Allison‐Logan , F. Karimi , N. J.‐A. Chan , W. Qiu , W. Duan , N. M. O'Brien‐Simpson , G. G. Qiao , Chem. Soc. Rev. 2020, 49, 4737–4834, 10.1039/C9CS00738E. 32573586

[13] O. C. Onder , P. Utroša , S. Caserman , M. Podobnik , E. Žagar , D. Pahovnik , Macromolecules 2021, 54, 8321–8330, 10.1021/acs.macromol.1c01490.

[14] J. Zhang , Q. Hu , S. Wang , J. Tao , M. Gou , Int. J. Bioprinting 2019, 6, 242, 10.18063/ijb.v6i1.242. PMC7415858 32782984

[15] N. Zivic , P. K. Kuroishi , F. Dumur , D. Gigmes , A. P. Dove , H. Sardon , Angew. Chem. Int. Ed. 2019, 58, 10410–10422, 10.1002/anie.201810118. 30575230

[16] A. Romano , I. Roppolo , E. Rossegger , S. Schlögl , M. Sangermano , Materials 2020, 13, 2777, 10.3390/ma13122777. 32575481 PMC7344511

[17] T. Stukenkemper , J. F. G. A. Jansen , C. Lavilla , A. A. Dias , D. F. Brougham , A. Heise , Polym. Chem. 2017, 8, 828–832.

[18] T. Stukenkemper , X. Paquez , M. W. G. M. Verhoeven , E. J. M. Hensen , A. A. Dias , D. F. Brougham , A. Heise , Macromol. Rapid Commun. 2018, 39, 1700743–1700748. 10.1002/marc.201700743 29333693

[19] D. Li , P. Chang‐Ming , ACS Macro Lett. 2017, 6, 292–297, 10.1021/acsmacrolett.7b00167. 35650905

[20] P. Li , Y. Song , C.‐M. Dong , Polym. Chem. 2018, 9, 3974–3986.

[21] S. L. Goodrich , M. R. Hill , R. A. Olson , B. S. Sumerlin , Polym. Chem. 2021, 12, 4104–4110.

[22] H. R. Kricheldorf , C. Von Lossow , G. Schwarz , J. Polym. Sci. Part Polym. Chem. 2006, 44, 4680–4695.

[23] Y. Wu , K. Chen , J. Wang , M. Chen , W. Dai , R. Liu , J. Am. Chem. Soc. 2024, 146, 24189–24208. 39172171 10.1021/jacs.4c05382

[24] E. Blasco , M. Wegener , C. Barner‐Kowollik , Adv. Mater. 2017, 29, 1604005, 10.1002/adma.201604005. 28075059

[25] P. Utroša , E. Žagar , D. Pahovnik , Eur. Polym. J. 2024, 204, 112707.

[26] E. Käpylä , T. Sedlačík , D. B. Aydogan , J. Viitanen , F. Rypáček , M. Kellomäki , Mater. Sci. Eng. C 2014, 43, 280–289, 10.1016/j.msec.2014.07.027. 25175215

[27] C. M. Viray , B. Van Magill , H. Zreiqat , Y. Ramaswamy , ACS Biomater. Sci. Eng. 2022, 8, 1115–1131. 35179029 10.1021/acsbiomaterials.1c01519

[28] R. D. Murphy , C. Delaney , S. Kolagatla , L. Florea , C. J. Hawker , A. Heise , Adv. Funct. Mater. 2023, 33, 2306710, 10.1002/adfm.202306710.

[29] R. D. Murphy , M. Cosgrave , N. Judge , E. Tinajero‐Diaz , G. Portale , B. Wu , A. Heise , Small 2024, 20, 2405578, 10.1002/smll.202405578. 39268774 PMC11618715

[30] J. F. Cameron , J. M. J. Frechet , J. Am. Chem. Soc. 1991, 113, 4303–4313, 10.1021/ja00011a038.

[31] Š. Gradišar , E. Žagar , D. Pahovnik , ACS Macro Lett. 2017, 6, 637–640. 35650850 10.1021/acsmacrolett.7b00379

[32] C. D. Vacogne , H. Schlaad , Chem. Commun. 2015, 51, 15645–15648, 10.1039/C5CC06905J. 26359317

[33] X. Liu , J. Huang , J. Wang , H. Sheng , Z. Yuan , W. Wang , W. Li , Z. Song , J. Cheng , CCS Chem. 2025, 7, 2769–2780.

[34] X. Zhang , W. Xi , C. Wang , M. Podgórski , C. N. Bowman , ACS Macro Lett. 2016, 5, 229–233, 10.1021/acsmacrolett.5b00923. 28018752 PMC5176105

[35] J. A. Vazquez , X. Lopez De Pariza , N. Ballinger , N. Sadaba , A. Y. Sun , A. O. Olanrewaju , H. Sardon , A. Nelson , Polym. Chem. 2025, 16, 589–597. 40271496 10.1039/d4py01120a PMC12014190

[36] M. Gaplovsky , Y. V. Il'ichev , Y. Kamdzhilov , S. V. Kombarova , M. Mac , M. A. Schwörer , J. Wirz , Photochem. Photobiol. Sci. 2005, 4, 33–42, 10.1039/b409927c. 15616689

[37] J. P. Menzel , B. B. Noble , J. P. Blinco , C. Barner‐Kowollik , Nat. Commun. 2021, 12, 1691, 10.1038/s41467-021-21797-x. 33727558 PMC7966369

[38] N. Zivic , T. Brossier , F. Crestey , S. Catrouillet , A. Chemtob , V. Héroguez , P. Lacroix‐Desmazes , C. Joly‐Duhamel , S. Blanquer , J. Pinaud , Prog. Org. Coat. 2022, 172, 107128, 10.1016/j.porgcoat.2022.107128.

[39] S. P. Rannard , N. J. Davis , Org. Lett. 2000, 2, 2117–2120, 10.1021/ol006020n. 10891244

[40] J. C. Foster , A. W. Cook , N. T. Monk , B. H. Jones , L. N. Appelhans , E. M. Redline , S. C. Leguizamon , Adv. Sci. 2022, 9, 2200770, 10.1002/advs.202200770. PMC9108613 35274480

