# Supplementary material for: Photoinduced Ring‐Opening Polymerization of N‐Carboxyanhydrides for the Preparation of Cross‐Linked Polypeptide Gels
Source: Angew Chem Int Ed Engl. 2025 Dec 18;65(6):e21891. doi: 10.1002/anie.202521891 (PMC12865261; doi:10.1002/anie.202521891)
Supplement: Supplementary file 1 — Supporting Information [file ANIE-65-e21891-s001.pdf]

Supporting Information

Photoinduced Ring-Opening Polymerization of *N*-Carboxyanhydrides for the Preparation of Cross-Linked Polypeptide Gels

Ana Kočman,[a,b] David Pahovnik,[a] Ema Žagar,[a] and Petra Utroša*[a]

[a] A. Kočman, Dr. D. Pahovnik, Dr. E. Žagar, Dr. P. Utroša
Department of Polymer Chemistry and Technology
National Institute of Chemistry
Hajdrihova 19, 1000 Ljubljana (Slovenia)
E-mail: petra.utrosa@ki.si

[b] A. Kočman
Faculty of Chemistry and Chemical Technology
University of Ljubljana
Večna pot 113, 1000 Ljubljana (Slovenia)

Contents

Experimental Section 1
Materials 1
Instrumentation 1
Synthesis 2
Linear polypeptides – ROP of BLG NCA 3
Gel preparation 3
Photomask experiments 4
Characterization Data 5
References 21

Figure S1. ^{1}H NMR spectrum with signal assignation for **BLG NCA** in DMSO-d_6 with added TFA. 5
Figure S2. ^{13}C NMR spectrum with signal assignation for **BLG NCA** in DMSO-d_6 with added TFA. 5
Figure S3. ^{1}H NMR spectrum with signal assignation for **(Boc-HCys-OH)$_2$** in DMSO-d_6 with added TFA. 6
Figure S4. ^{1}H NMR spectrum with signal assignation for **HCys NCA** in DMSO-d_6 with added TFA. 6
Figure S5. ^{13}C NMR spectrum with signal assignation for **HCys NCA** in DMSO-d_6 with added TFA. 7
Figure S6. ^{1}H NMR spectrum with signal assignation for 2-nitrobenzyl dibutylcarbamate (**PB-DBA)** in DMSO-d_6 with added TFA. 7
Figure S7. ^{13}C NMR spectrum with signal assignation for 2-nitrobenzyl dibutylcarbamate (**PB-DBA)** in DMSO-d_6..8
Figure S8. ^{1}H NMR spectrum with signal assignation for 2-nitrobenzyl tetramethylguanidinecarbamate (**PB-TMG)** in DMSO-d_6 with added TFA. 8
Figure S9. ^{13}C NMR spectrum with signal assignation for 2-nitrobenzyl tetramethylguanidinecarbamate (**PB-TMG)** in DMSO-d_6..9
Figure S10. Absorption spectra of BLG NCA (10^{-3} M), HCys NCA (10^{-3} M), PB-DBA (10^{-4} M), PB-TMG (10^{-4} M) and DBA (10^{-2} M) in DMF versus the emission spectrum of UV-light source used. 11
Figure S11. Monomer conversion as a function of reaction time (1.0 M BLG NCA in anhydrous DMF with 1.5 % PB-DBA or 1.5% PB-TMG) in the absence of irradiation..............................11
Figure S12. Absorption spectra of PB-DBA (0.01 M in DMF) over 10 min of irradiation at 365 nm. The images in the black and pink rectangles show the colour change of the 0.01 M solution of PB-DBA in DMF from transparent (before irradiation) to yellow (after 10 min irradiation).......12
Figure S13. ^{1}H NMR spectra of BLG NCA without (photo)catalyst in anhydrous DMF with TFA (0.50 mol % relative to NCA): **A**) before irradiation and **B**) after 10 min irradiation (365 nm, 90

mW·cm^{-2}), followed by 50 min in the absence of light. The spectra were recorded in DMSO-d_6 with added TFA. 12
Figure S14. SEC/MALS-RI chromatograms of polypeptides prepared by photo-ROP (3.0 % PB-DBA) and by ROP (1.2 % DBA). The solid and dashed curves in the SEC chromatograms represent the RI and 90° LS detector responses, respectively, while the dotted lines show the molar mass as a function of elution volume. 13
Figure S15. Gradual release of DBA from PB-DBA over time during 10 min of irradiation at different initial PB-DBA concentrations: **A**) The amount of DBA released relative to BLG NCA; **B**) PB-DBA cleavage conversion over time. 13
Figure S16. Gradual release of DBA from PB-DBA over time during 10 min of irradiation at different light intensities, showing the increasing amount of DBA released: **A**) The amount of DBA released relative to BLG NCA; **B**) PB-DBA cleavage conversion over time. 14
Figure S17. Time-dependent oscillatory rheological test showing light-triggered gelation of P(BLG-co-HCys), with gel points consistently appearing approximately 14 min after the onset of irradiation, regardless of whether the light was applied after 5 or 15 min. Illumination periods are depicted with violet or grey rectangle. 14
Figure S18. Monomer conversion as a function of time for co-ROP of BLG NCA and HCys NCA: Photo-ROP with PB-DBA under 10 min of irradiation, and with DBA without irradiation. 15
Figure S19. ^{1}H NMR spectra of HCys NCA in anhydrous DMF (0.08 M) with TFA (6.25 % relative to HCys NCA): **A**) before irradiation; **B**) after 60 min in the absence of light; **C)** after 10 min of irradiation (365 nm, 90 mW·cm^{-2}), followed by 50 min in the absence of light. The spectra were recorded in DMSO-d_6 with added TFA. 15
Figure S20. Time-dependent oscillatory test results for the photoinduced ring-opening copolymerization of BLG NCA and HCys NCA: **A**) at different light intensities; **B**) at different PB-DBA initial concentrations. 16
Figure S21. **A**) Gel content and **B**) swelling degree values of P(BLG-co-HCys) gels at different initial concentrations of photobase, at different light intensities and type of (photo)base. 17
Figure S22. Set-up of a photomask experiment, showing irradiation of a sample from above. The sample was covered with a glass slide, which was shielded by a UV-impermeable plate of optional shape. 18
Figure S23. **A**) Schematic representation of the half-irradiated sample in the photomask experiment, showing sampling points for FTIR being approximately 7 mm apart. Positions -2 and -1 indicate the non-irradiated side, position 0 is the centre, and positions 1 and 2 are the irradiated parts of the reaction mixture; **B**) Monomer conversion as a function of position for photo-ROP after 12 min irradiation, **with (+)** and **without (-)** TFA; **C**) FTIR spectra recorded at different positions of the reaction mixture (**with (+) TFA**) after 12 min of irradiation in the photomask experiment; **D**) FTIR spectra recorded at different positions of the reaction mixture (**without (-) TFA**) after 12 min of irradiation in the photomask experiment. 19
Figure S24: Optical microscopy image of the tip of an arm of the star-shaped gel at 40× magnification. 20

Table S1. Experimental conditions for linear and cross-linked polypeptide preparation. 10

Experimental Section

Materials

γ-Benzyl-L-glutamate (BLG) and L-homocystine (HCys) were purchased from Iris Biotech GmbH. 2-Nitrobenzyl alcohol, acetic acid, anhydrous ethyl acetate, anhydrous *N*,*N*-dimethylformamide (DMF), anhydrous tetrahydrofuran (THF), carbonyldiimidazole (CDI), chloroform, dibutylamine (DBA), dichloromethane, di-*tert*-butyl-dicarbonate (Boc_2O), lithium bromide (LiBr), magnesium sulphate ($MgSO_4$), potassium bisulphate ($KHSO_4$), sodium chloride (NaCl), 1,1,3,3-tetramethylguanidine (TMG) and triphosgene were purchased from Sigma Aldrich. Dioxane, hydrochloric acid (HCl), methanol, *N*,*N*-dimethylacetamide (DMAc), *n*-hexane, potassium carbonate (K_2CO_3), potassium hydroxide (KOH) and THF were purchased from Merck. Anhydrous toluene was purchased from Merck Aldrich. Trifluoroacetic acid (TFA) was obtained from Acros Organics. Dimethyl sulfoxide-d_6 (DMSO-d_6) was purchased from Eurisotop.

Instrumentation

^1H and ^{13}C nuclear magnetic resonance (NMR) spectra were recorded on a Bruker 600 MHz spectrometer (Bruker Corporation, USA). All measurements were carried out at room temperature in DMSO-d_6 with tetramethylsilane (TMS, $\delta = 0$) as internal reference standard. If necessary, a few drops of trifluoroacetic acid were added.

Fourier transform infrared (FTIR) spectra were recorded in attenuated total reflectance (ATR) mode in the spectral range of 400–4500 cm^{-1}using the Spectrum-Two FTIR spectrometer (Perkin Elmer, U.K.).

For the irradiation experiments, we used a UV (365 nm) mounted LED (M365L3) equipped with an adjustable collimation adapter (SM1U) and connected to a driver (LEDD1B). The light intensity was measured with a thermal power sensor (S405C) connected to a power meter (PM100D). All equipment was purchased from Thorlabs, Inc. USA.

The molar mass characteristics (M_w, M_n, dispersity: $Đ = M_w/M_n$) of the precipitated polypeptides after complete monomer conversion were determined by SEC connected to a DAWN multi-angle light scattering (MALS) photometer (Wyatt Technology Corp., USA), and an Optilab interferometric refractometer (RI) (Wyatt Technology Corp., USA) as the concentration detector. Separations were performed at 50 °C using a PolarGel M column (7.5 mm × 300.0 mm, pore size 8 μm, Agilent Technologies) with a precolumn. 0.05 M solution of LiBr in DMAc at a flow rate of 0.8 $mL \cdot min^{-1}$ was used as the solvent and mobile phase. The mass of the samples injected onto the column was typically $1.0 \cdot 10^{-3}$ g, whereas the concentration of the solutions was $1.0 \cdot 10^{-3}$ $g \cdot mL^{-1}$. The sample d*n*/d*c* value, required for calculation of molar mass characteristics, was determined assuming 100 % mass recovery of the sample from the column. Astra 8.2.0. software (Wyatt Technology Corp., USA) was used for data acquisition and evaluation.

UV-visible (UV-vis) spectrometry was performed with a NANOCOLOR UV-VIS/II spectrometer (Macherey-Nagel, GmbH & Co. KG, Germany). The spectra were recorded from 190 to 900 nm

using a quartz cuvette with a 10 mm path length. The samples were dissolved in DMF at different concentrations.

Rheological measurements of gels were performed on a MCR 302 rheometer (Anton Paar, Austria) equipped with parallel plate measuring system (PP25, Anton Paar, Austria) with a diameter of 25 mm and a gap of 0.60 or 0.70 mm. All experiments were conducted at 20 °C. A time-dependent oscillatory test was performed with a constant shear strain of 0.5 % and a frequency of 10 Hz. The reaction mixtures were placed on a lower quartz glass plate and the irradiation was externally controlled from below. The light intensity was measured with a power meter. The gel point was determined as the crossover point of storage (*G*') and loss (*G*'') modulus.

The compressive mechanical properties of the gel samples were evaluated using a DMA Q800 dynamic mechanical analyzer (TA Instruments, USA) equipped with 40 mm diameter compression discs. A preload force of 0.05 N was applied to ensure proper contact between the sample and the discs. The specimens were compressed to failure at a strain rate of 25 %·min^{-1}. The compressive moduli were calculated as the slope of the stress-strain curve within the linear initial range. Each measurement was performed in triplicate and the results are reported as mean values with standard deviations.

Optical microscopy was performed using Traveler optical microscope (Supra Foto Elektronik Vertriebs GmbH, Germany) at 40× magnification.

Synthesis

γ-Benzyl-L-glutamate *N*-carboxyanhydride (BLG NCA) was synthesized according to a previously established procedure.[1] The ^{1}H and ^{13}C NMR spectra with signal assignation are shown in Figures S1 and S2.

***N*,*N*'-Di-(tert-butyloxycarbonyl)-L-homocystine ((Boc-Hcys-OH)$_2$)** and **L-homocystine *N*-carboxyanhydride (HCys NCA)** were prepared as previously reported.[2] The ^{1}H NMR spectrum of (BocHcysOH)$_2$ with signal assignation is shown in Figure S3. The ^{1}H and ^{13}C NMR spectra of HCys NCA with signal assignation are shown in Figures S4 and S5.

2-Nitrobenzyl dibutylcarbamate (PB-DBA) was synthesized by methods based on other 2-nitrobenzyl carbamate-based photobases.[3,4] 2-Nitrobenzyl alcohol (2.50 g, 16.3 mmol) and KOH (0.023 g, 0.41 mmol) were dissolved in 60 mL of anhydrous toluene, followed by the addition of CDI (2.91 g, 18.0 mmol), after which a precipitate was formed. The reaction was stirred for 1.5 h at 60 °C. DBA (29.5 g, 288.5 mmol) was added slowly and the solution was stirred for a further 3 h at 60 °C. The precipitate dissolved about 30 min after the addition of DBA. The reaction mixture was concentrated in vacuo and diluted with dichloromethane (60 mL). The product was extracted with 5 % HCl solution (2 x 60 mL) and brine (60 mL), dried over $MgSO_4$ and filtered. The solvent was removed under vacuum. The product was further purified on a silica gel column eluting with a mixture of hexane and ethyl acetate in a 3:1 ratio (R_f = 0.44). The purified product was obtained in the form of a yellow liquid (2.80 g, 51 % yield). The ^{1}H and ^{13}C spectra with signal assignation are shown in Figures S6 and S7.

2-Nitrobenzyl tetramethylguanidinecarbamate (PB-TMG) was synthesized according to a previously reported procedure.[4] The ^{1}H and ^{13}C spectra with signal assignation are shown in Figures S8 and S9.

Linear polypeptides – ROP of BLG NCA

In a typical experiment, BLG NCA (0.158 g, 0.70 mmol) was dissolved in anhydrous DMF (495 µL). A solution of TFA in anhydrous DMF (15 µL, 0.2 M) and a solution of PB-DBA in anhydrous DMF (90 µL, 0.2 M) were added. The experimental conditions for all experiments are summarized in Table S1. The reaction mixture was transferred to a glass vial (Macherey-Nagel, 8.2 mm × 40 mm, 1.0 mL) and irradiated at 365 nm (90 mW·cm^{-2}) for 10 min. The reaction mixture was allowed to polymerize at room temperature until complete monomer conversion, which was confirmed by FTIR or ^{1}H NMR. For ^{1}H NMR, samples were aliquoted at different times (20 µL), diluted in DMSO-d_6 and quenched with a solution of acetic acid in DMSO-d_6 (25 µL, 0.1 M). Monomer conversions were calculated from signal areas obtained from ^{1}H NMR spectra according to Equation (S1):

$$\chi\,(\%) = \frac{A_{5.21} - 2 \cdot A_{4.47}}{A_{5.21}} \cdot 100 \qquad \text{(S1)}$$

where χ represents the conversion of BLG NCA. $A_{5.21}$ is the signal area corresponding to the methylene protons (-CH_2-) present in both monomer and polymer, while $A_{4.47}$ is the signal area corresponding to the methyne protons (-CH-) of monomer.

The remaining reaction mixture was then poured into cold MQ water to precipitate the product. The precipitate was isolated by centrifugation (8000 rpm, 4 min), washed twice with cold MQ water and dried in vacuo for 24 h.

Gel preparation

For the preparation of gels, BLG NCA (0.146 g, 0.55 mmol) and HCys NCA (0.015 g, 0.048 mmol) were dissolved in anhydrous DMF (495 µL). A solution of TFA in anhydrous DMF (15.0 µL, 0.2 M) and a solution of PB-DBA in anhydrous DMF (90 µL, 0.2 M) were added. Thus obtained solution was transferred to a plastic mould, which was sealed with a needle-pierced septum to allow the release of CO_2 formed during the reaction. The solution was irradiated at 365 nm (90 mW·cm^{-2}) for 10 min. The experimental conditions for each experiment are listed in Table S1. Polymerization was carried out for 24 h at room temperature and complete monomer conversion was confirmed by FTIR. Gels were then immersed in DMF at room temperature and removed at specific time intervals until their weight remained constant. Before weighing, the DMF on the surface of swollen samples was removed with filter paper. The solvent was removed by Soxhlet extraction with 1,4-dioxane for 24 h, followed by freeze drying. The degree of swelling was calculated according to Equation (S2):

$$\mathit{Swelling\ degree}\,(\%) = \frac{m_{\mathrm{s}} - m_{\mathrm{d}}}{m_{\mathrm{d}}} \cdot 100 \qquad \text{(S2)}$$

where m_{s} and m_{d} are the weights of the swollen gel and the dried residue, respectively.

The gel content was determined by dividing the weight of the dry gel by the theoretical weight of the polypeptide in the gel, assuming complete conversion of the monomers and accounting for CO_2 release, as shown in the Equation (S3):

$$Gel\ content\ (\%) = \frac{m_d}{m_{\mathrm{BLG\ NCA}} \cdot \left(1 - \frac{M_{\mathrm{CO_2}}}{M_{\mathrm{BLG\ NCA}}}\right) + m_{\mathrm{HCys\ NCA}} \cdot \left(1 - \frac{2 \cdot M_{\mathrm{CO_2}}}{M_{\mathrm{HCys\ NCA}}}\right)} \cdot 100 \quad \text{(S3)}$$

where $m_{\mathrm{BLG\ NCA}}$ and $m_{\mathrm{HCys\ NCA}}$ represent the weights of BLG NCA and HCys NCA in the gel, respectively. $M_{\mathrm{BLG\ NCA}}$ and $M_{\mathrm{HCys\ NCA}}$ correspond to the molar masses of BLG NCA (263.25 $g \cdot mol^{-1}$) and HCys NCA (292.28 $g \cdot mol^{-1}$), respectively. M_{CO2} represents the molar mass of CO_2 (44.01 g mol^{-1}).

Photomask experiments

The reaction mixture for gel preparation (1 M NCA, BLG NCA:HCys NCA = 92:8, 3.0 % PB-DBA; 0.55 mL) was transferred to a glass petri dish (30 mm diameter) and covered with a glass plate, partially shielded with UV-impermeable material to serve as a simple photomask. The solution was irradiated for 12 min from above with a 365 nm LED (60 $mW \cdot cm^{-2}$). After irradiation, the gel / liquid was analyzed by FTIR at the non-irradiated side, at the central point and at the irradiated side. Sampling and measurements were completed after 10 min.

Characterization Data

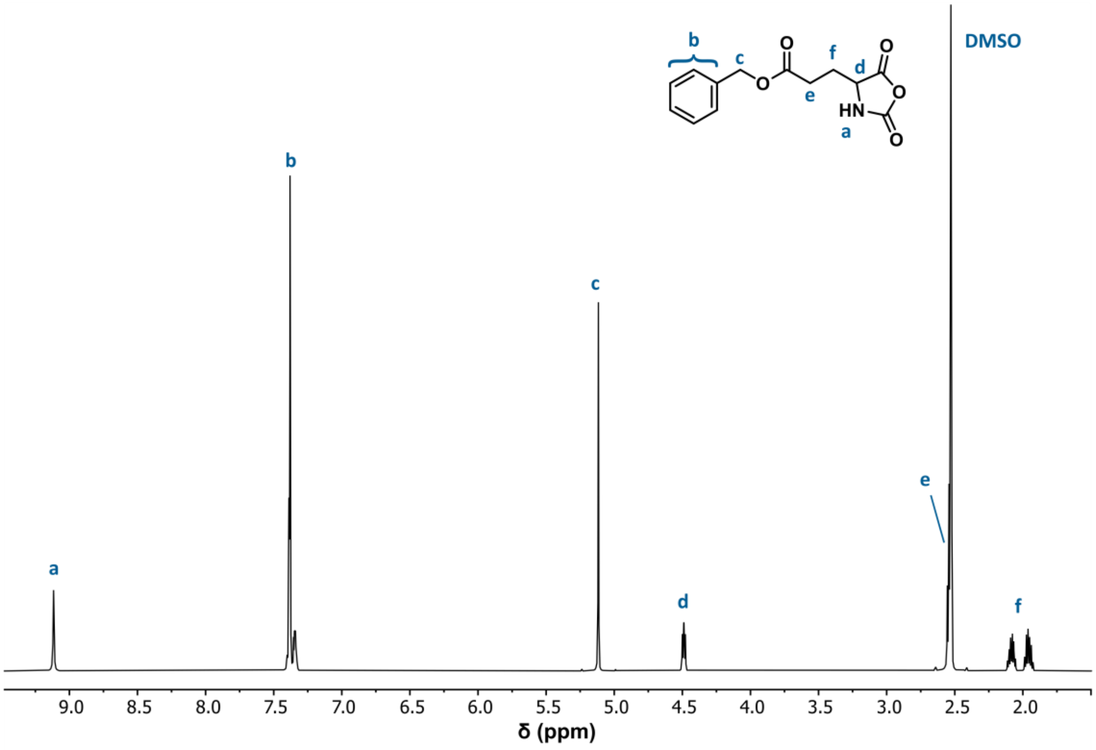

Figure S1. ^{1}H NMR spectrum with signal assignation for **BLG NCA** in DMSO-d_6 with added TFA.

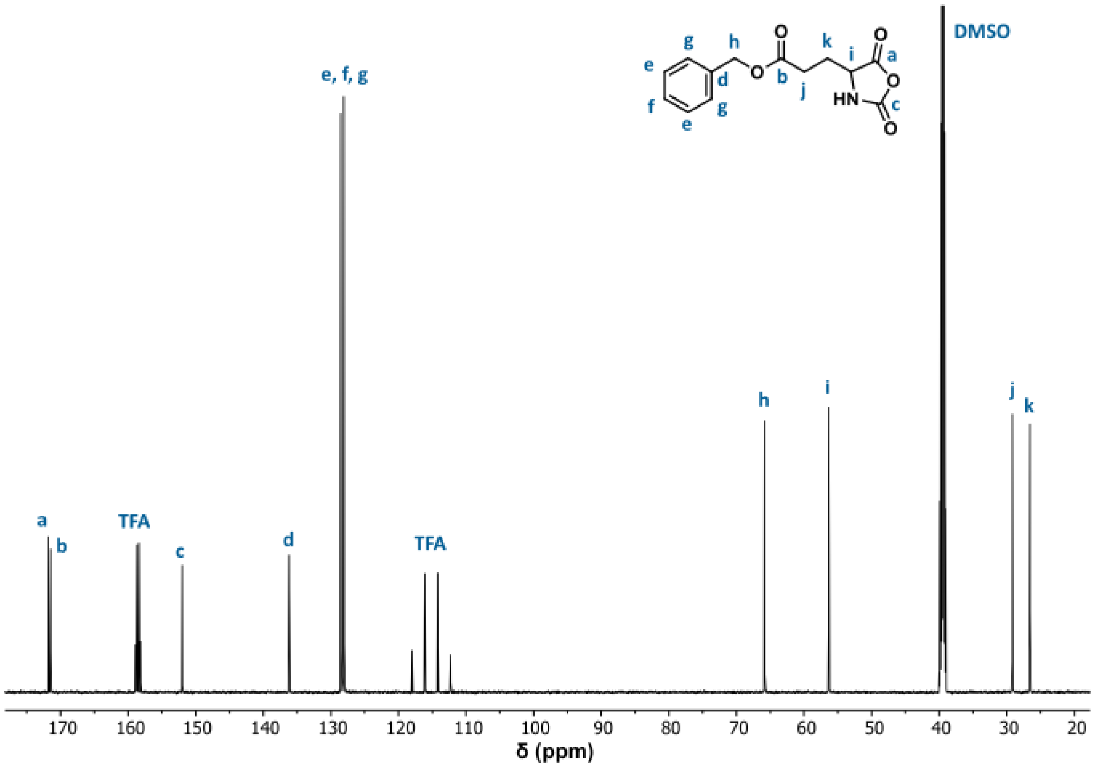

Figure S2. ^{13}C NMR spectrum with signal assignation for **BLG NCA** in DMSO-d_6 with added TFA.

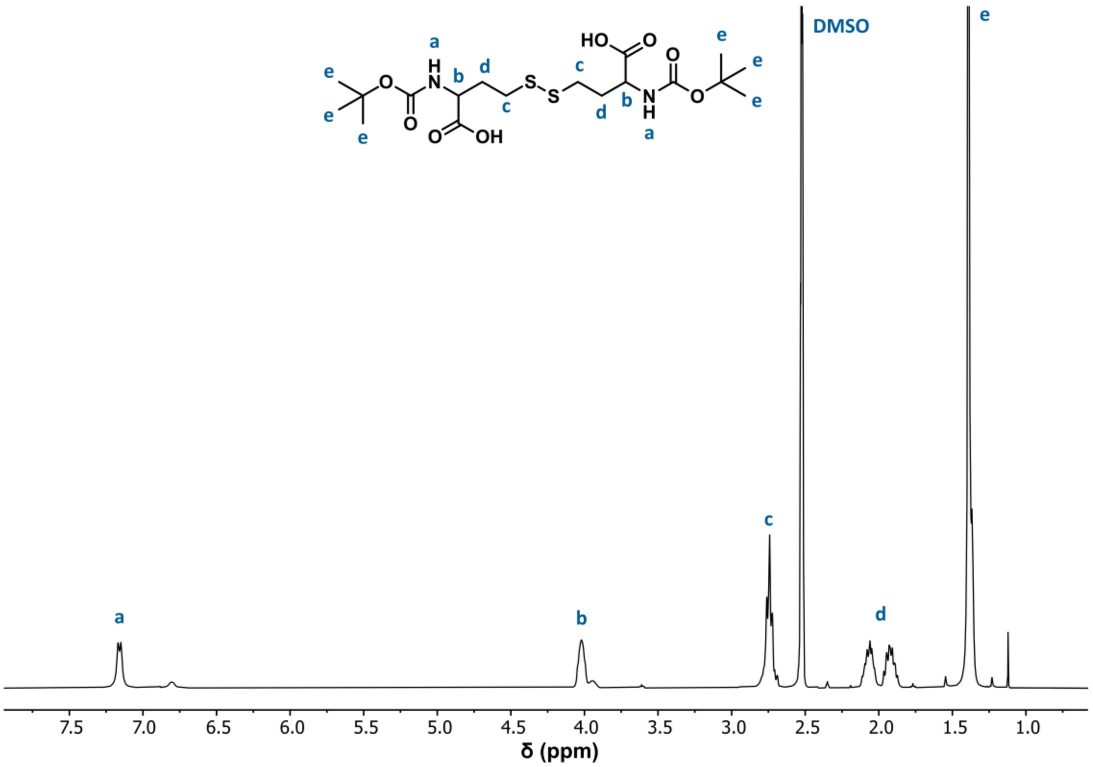

Figure S3. ^{1}H NMR spectrum with signal assignation for **(Boc-HCys-OH)₂** in DMSO-d_6 with added TFA.

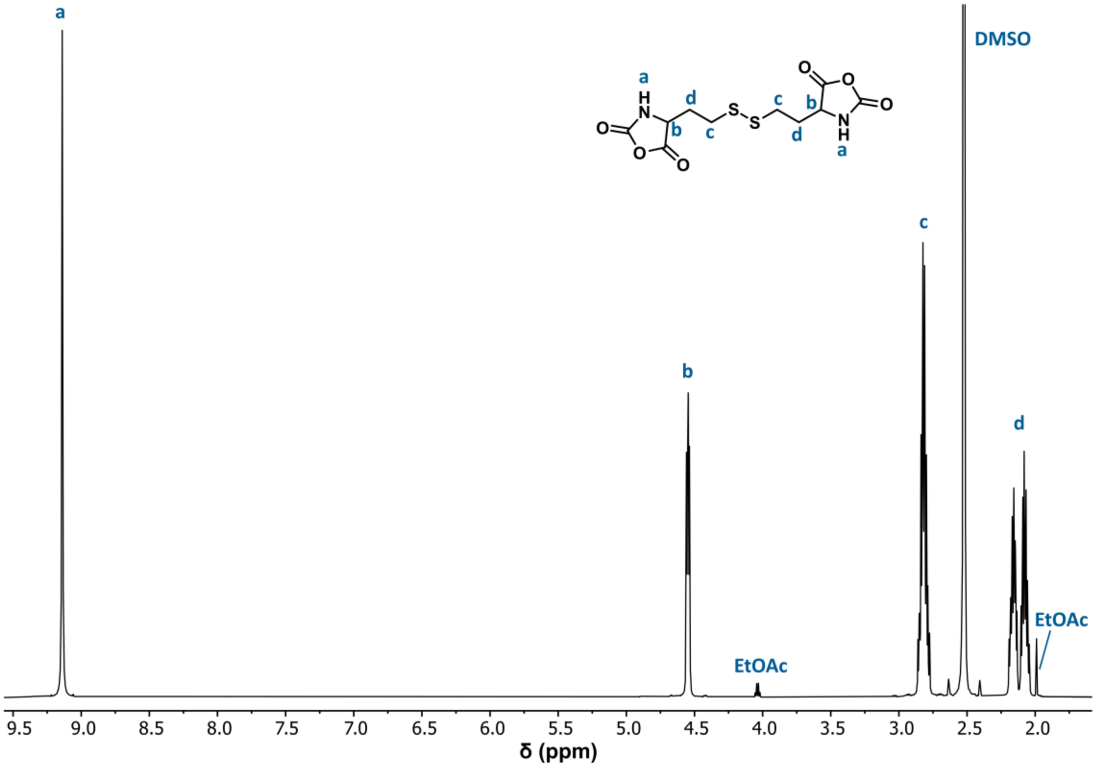

Figure S4. ^{1}H NMR spectrum with signal assignation for **HCys NCA** in DMSO-d_6 with added TFA.

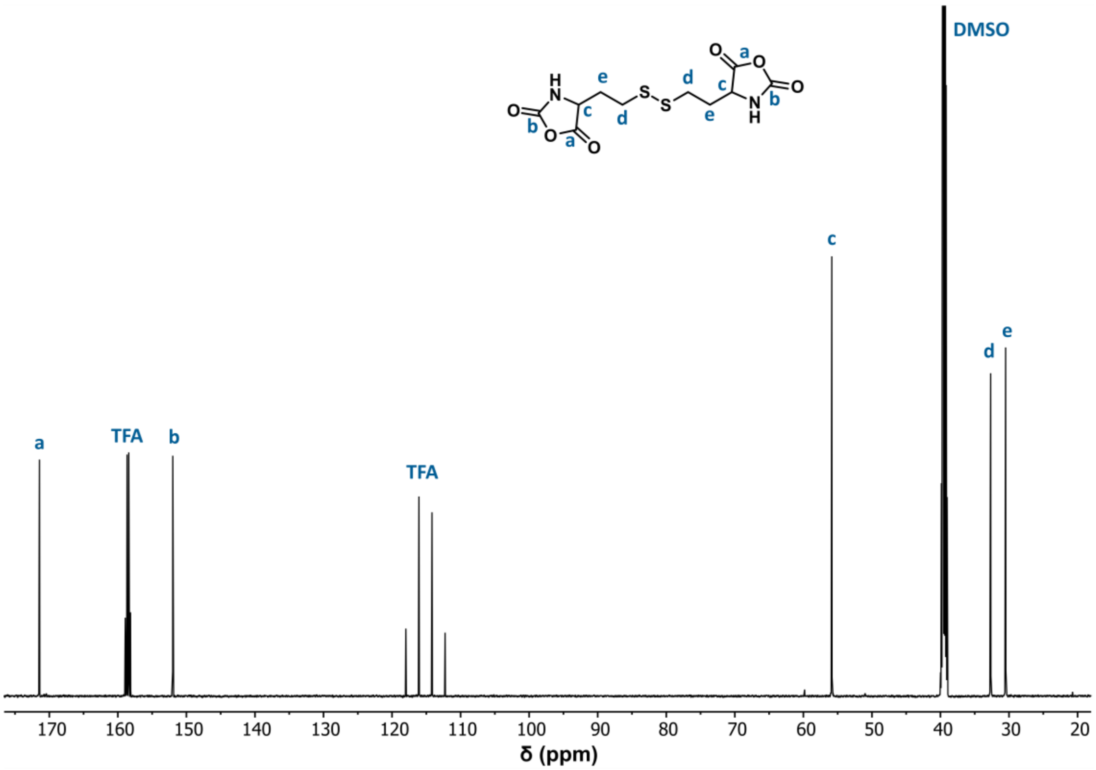

Figure S5. ^{13}C NMR spectrum with signal assignation for **HCys NCA** in DMSO-d_6 with added TFA.

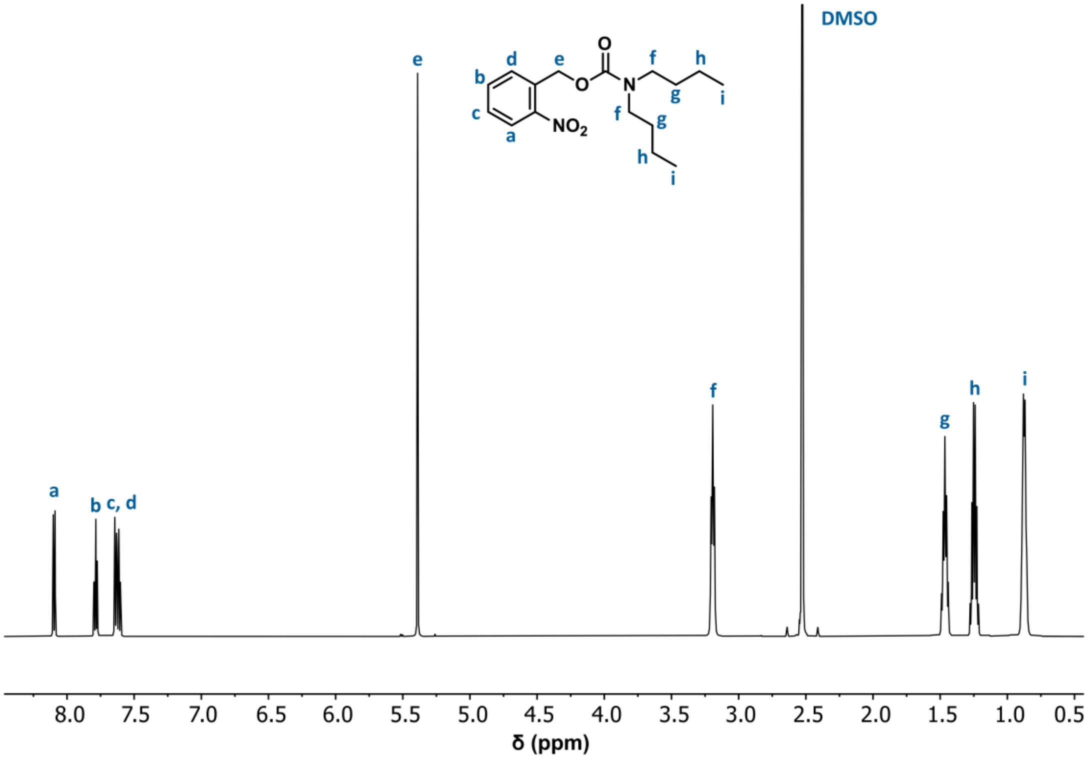

Figure S6. ^{1}H NMR spectrum with signal assignation for 2-nitrobenzyl dibutylcarbamate (**PB-DBA)** in DMSO-d_6 with added TFA.

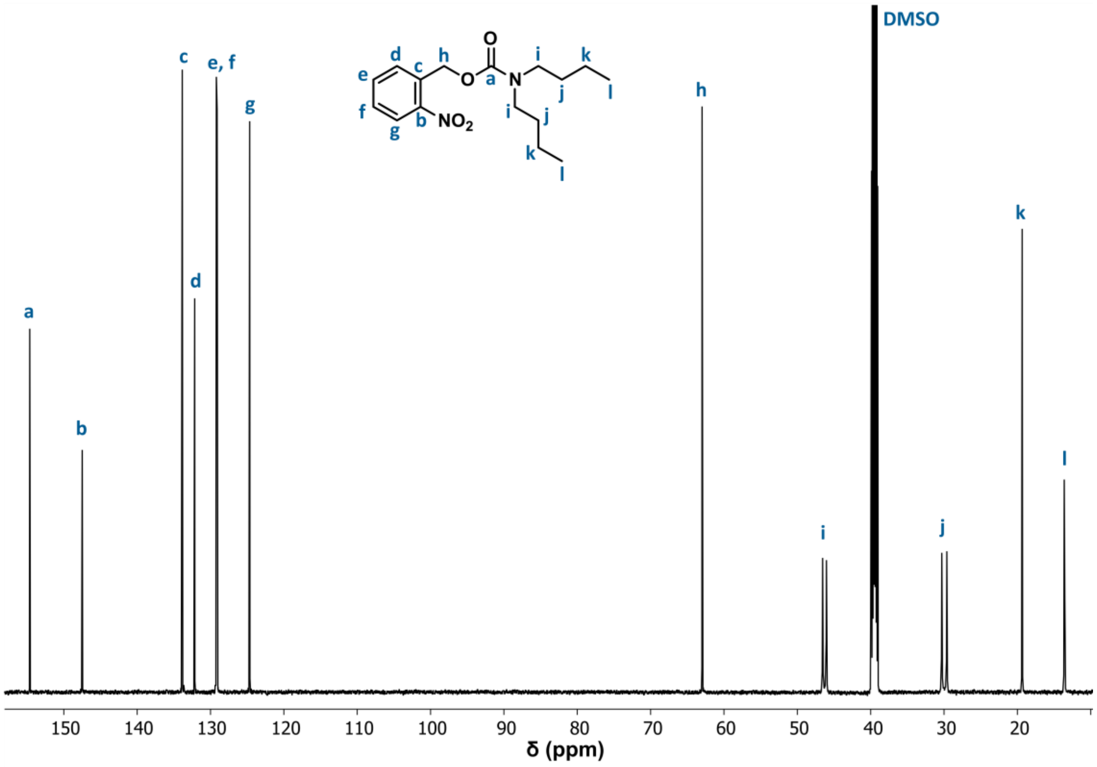

Figure S7. ^{13}C NMR spectrum with signal assignation for 2-nitrobenzyl dibutylcarbamate (**PB-DBA)** in DMSO-d_6.

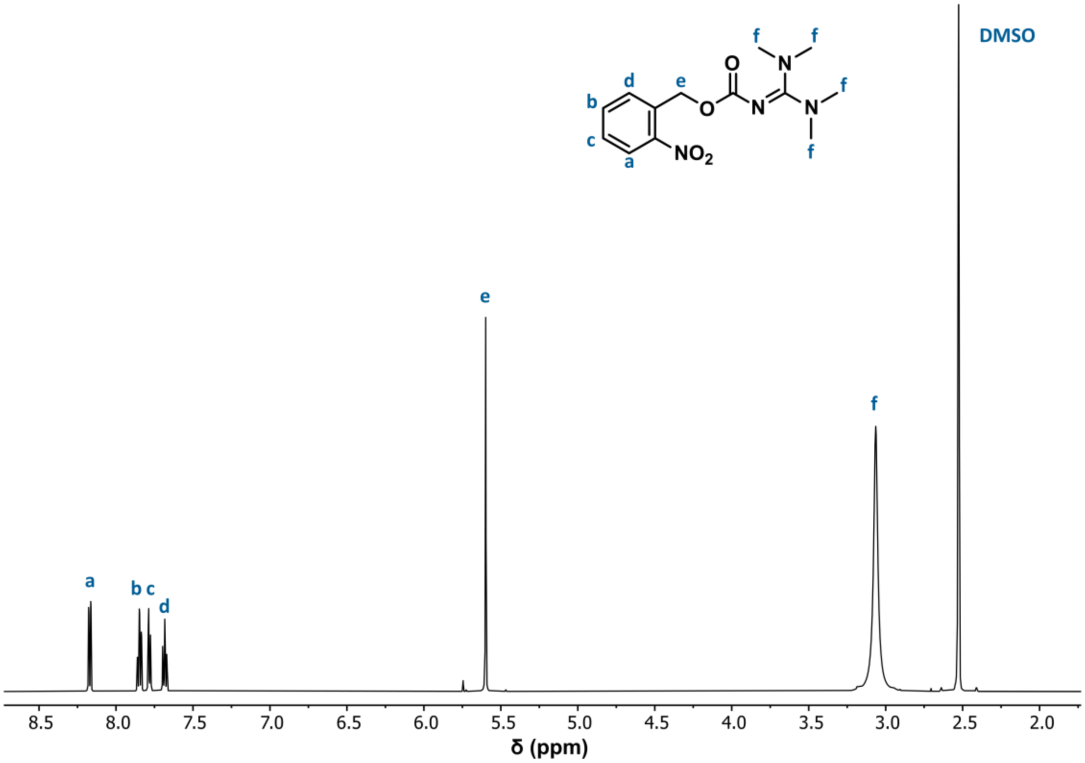

Figure S8. ^{1}H NMR spectrum with signal assignation for 2-nitrobenzyl tetramethylguanidinecarbamate (**PB-TMG)** in DMSO-d_6 with added TFA.

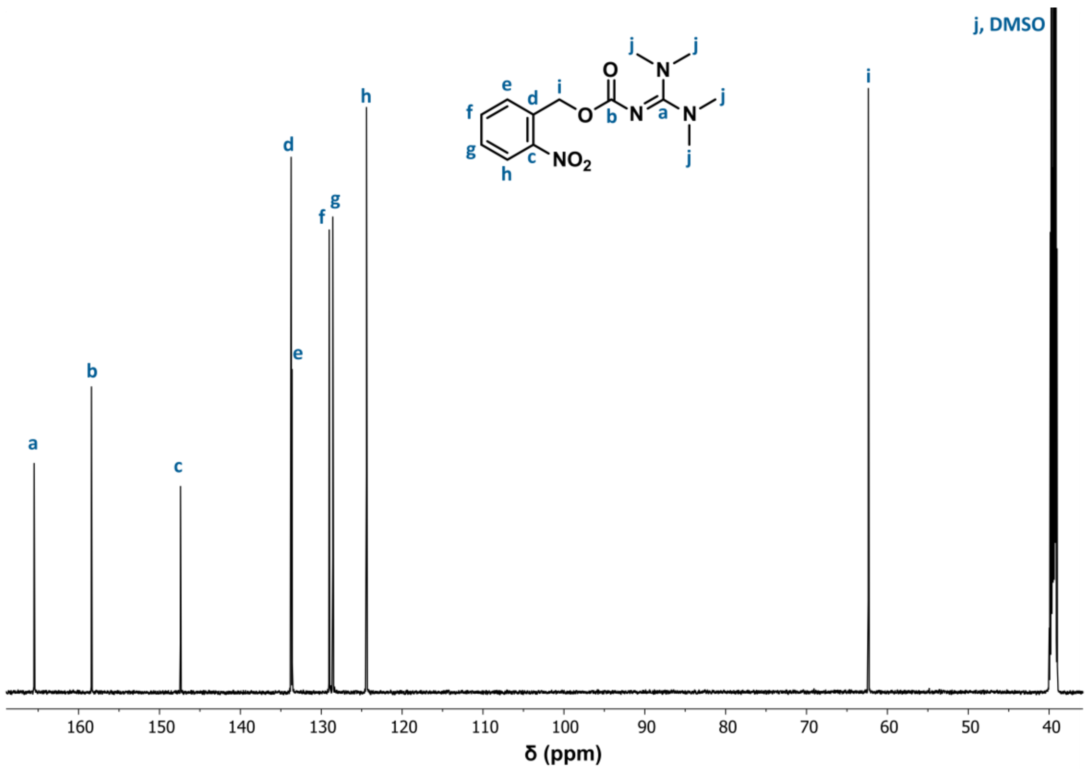

Figure S9. ^{13}C NMR spectrum with signal assignation for 2-nitrobenzyl tetramethylguanidinecarbamate (**PB-TMG)** in DMSO-d_6.

Table S1. Experimental conditions for linear and cross-linked polypeptide preparation.

Entry	*m* (BLG NCA) (mg)	*m* (HCys NCA) (mg)	Catalyst	Light intensity (mW·cm^{-2})	*x* (catalyst) (mol %) [a]	*V* (solvent) (μL) [b]	*V* (catalyst) (μL) [c]
				Linear			
1	158	None	None	90	0.0	600	0
2	158	None	DBA	Not irr.	1.2	575	24
3	158	None	PB-TMG	90	1.5	510	90
4	158	None	PB-DBA	90	0.5	585	15
5	158	None	PB-DBA	90	1.5	555	45
6	158	None	PB-DBA	90	3.0	510	90
7	158	None	PB-DBA	90	6.0	420	180
8	158	None	PB-DBA	45	3.0	510	90
9	158	None	PB-DBA	15	3.0	510	90
				Cross-linked			
10	146	15.4	None	90	0.0	600	0
11	146	15.4	DBA	Not irr.	1.2	575	24
12	146	15.4	PB-DBA	90	0.5	585	15
13	146	15.4	PB-DBA	90	1.5	555	45
14	146	15.4	PB-DBA	90	3.0	510	90
15	146	15.4	PB-DBA	90	6.0	420	180
16	146	15.4	PB-DBA	45	3.0	510	90
17	146	15.4	PB-DBA	15	3.0	510	90
18[d]	194	20.5	DBA	Not irr.	2.5	730	70

[a] The amount of catalyst is relative to the amount of NCAs.

[b] Solvent is anhydrous DMF with added TFA. The amount of TFA in DMF is 0.50 mol % relative to the NCAs.

[c] Catalyst was added as a solution in anhydrous DMF: 0.2 M for PB-DBA, 0.1 M for PB-TMG, 0.3 M for DBA.

[d] Conditions for the rheology experiment with DBA.

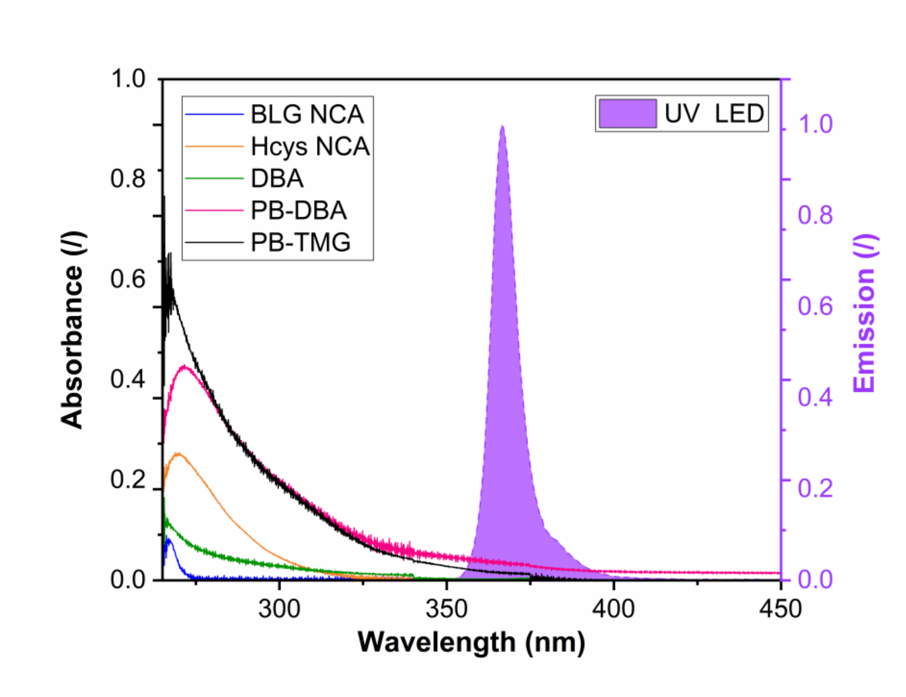

Figure S10. Absorption spectra of BLG NCA (10^{-3} M), HCys NCA (10^{-3} M), PB-DBA (10^{-4} M), PB-TMG (10^{-4} M) and DBA (10^{-2} M) in DMF versus the emission spectrum of UV-light source used.

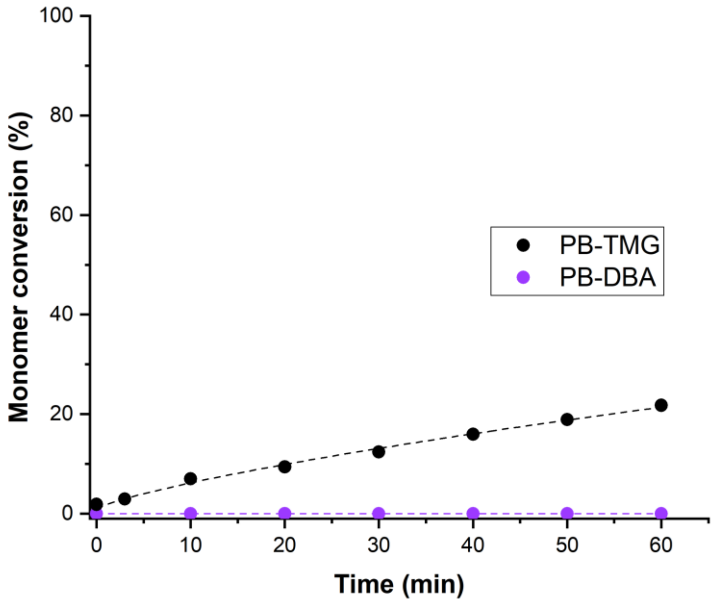

Figure S11. Monomer conversion as a function of reaction time (1.0 M BLG NCA in anhydrous DMF with 1.5 % PB-DBA or 1.5% PB-TMG) in the absence of irradiation.

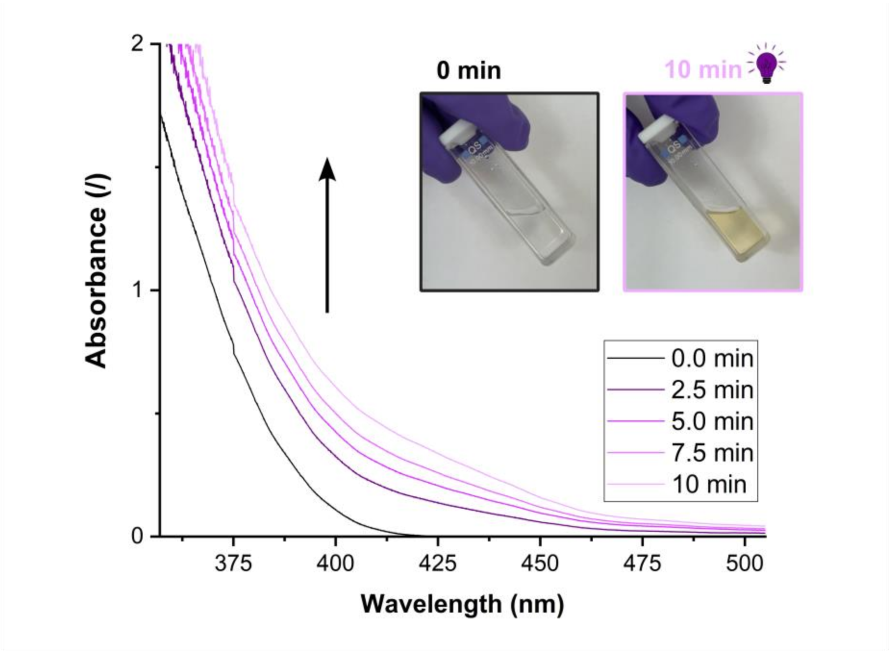

Figure S12. Absorption spectra of PB-DBA (0.01 M in DMF) over 10 min of irradiation at 365 nm. The images in the black and pink rectangles show the colour change of the 0.01 M solution of PB-DBA in DMF from transparent (before irradiation) to yellow (after 10 min irradiation).

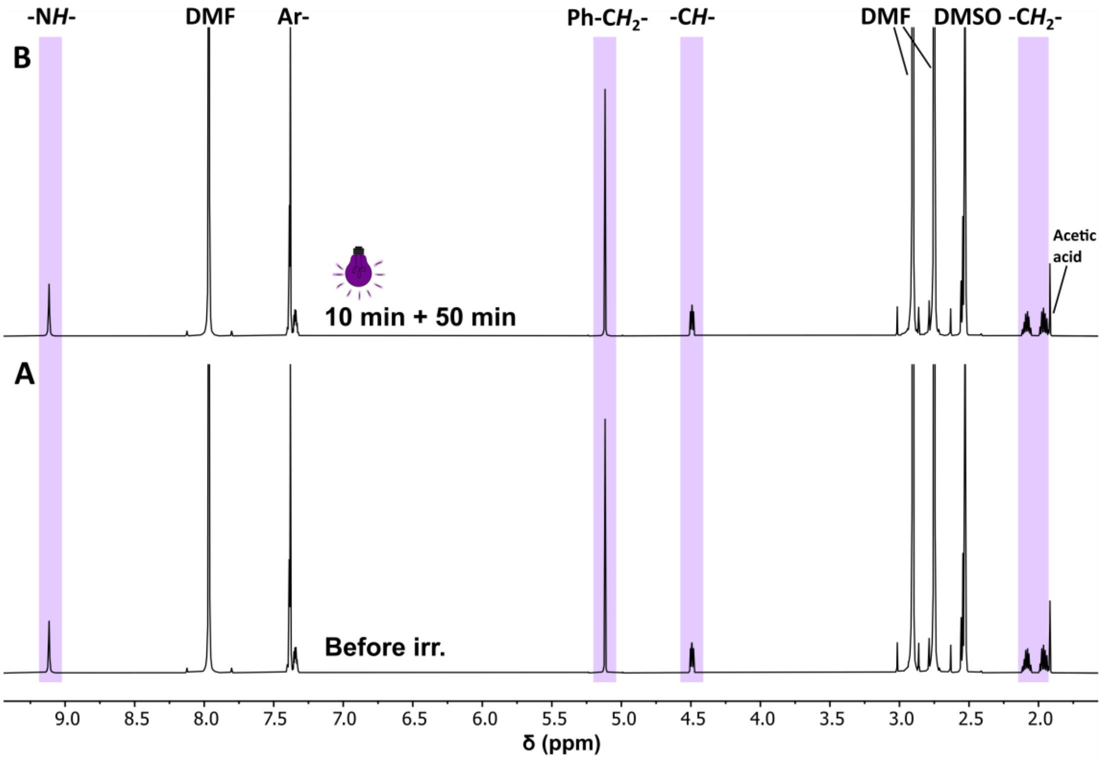

Figure S13. ^{1}H NMR spectra of BLG NCA without (photo)catalyst in anhydrous DMF with TFA (0.50 mol % relative to NCA): **A**) before irradiation and **B**) after 10 min irradiation (365 nm, 90 mW·cm^{-2}), followed by 50 min in the absence of light. The spectra were recorded in DMSO-d_6 with added TFA.

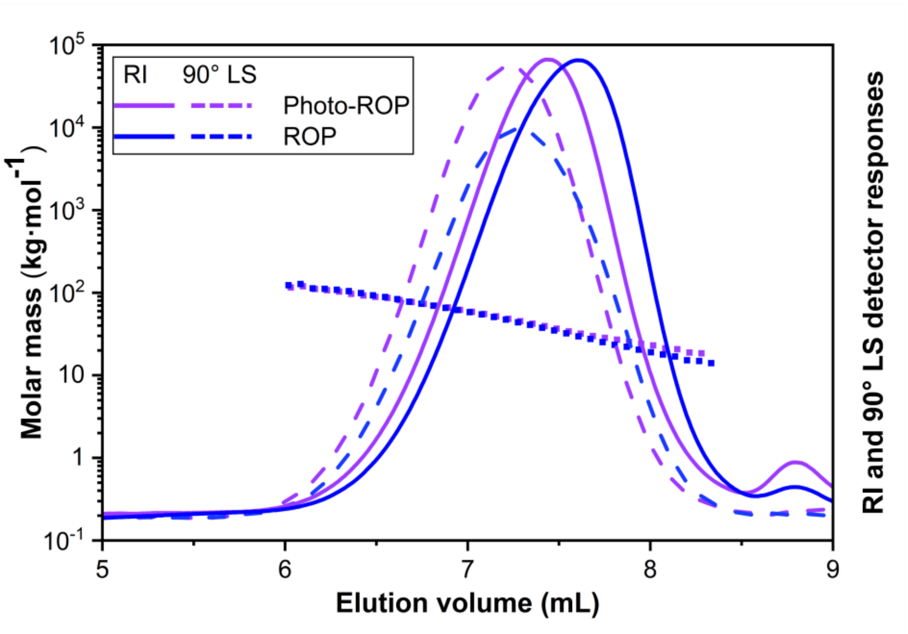

Figure S14. SEC/MALS-RI chromatograms of polypeptides prepared by photo-ROP (3.0 % PB-DBA) and by ROP (1.2 % DBA). The solid and dashed curves in the SEC chromatograms represent the RI and 90° LS detector responses, respectively, while the dotted lines show the molar mass as a function of elution volume.

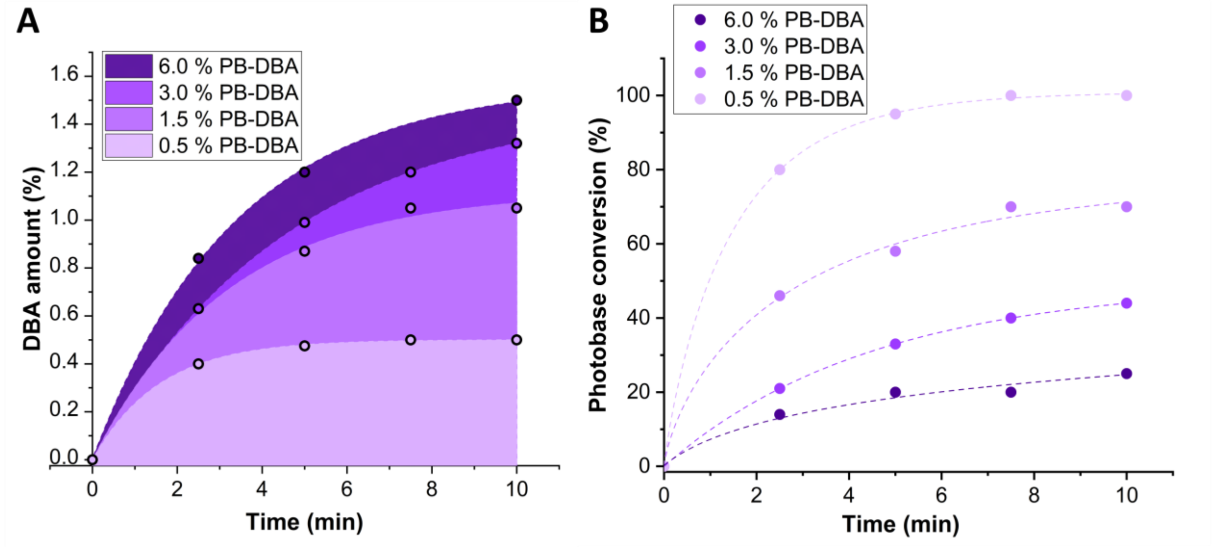

Figure S15. Gradual release of DBA from PB-DBA over time during 10 min of irradiation at different initial PB-DBA concentrations: **A**) The amount of DBA released relative to BLG NCA; **B**) PB-DBA cleavage conversion over time.

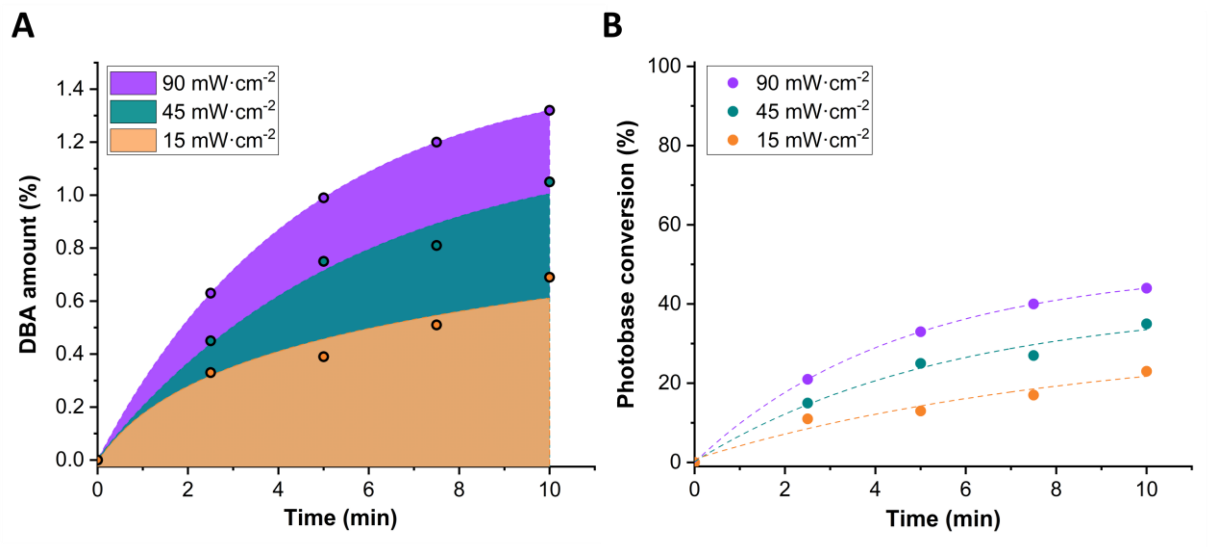

Figure S16. Gradual release of DBA from PB-DBA over time during 10 min of irradiation at different light intensities, showing the increasing amount of DBA released: **A**) The amount of DBA released relative to BLG NCA; **B**) PB-DBA cleavage conversion over time.

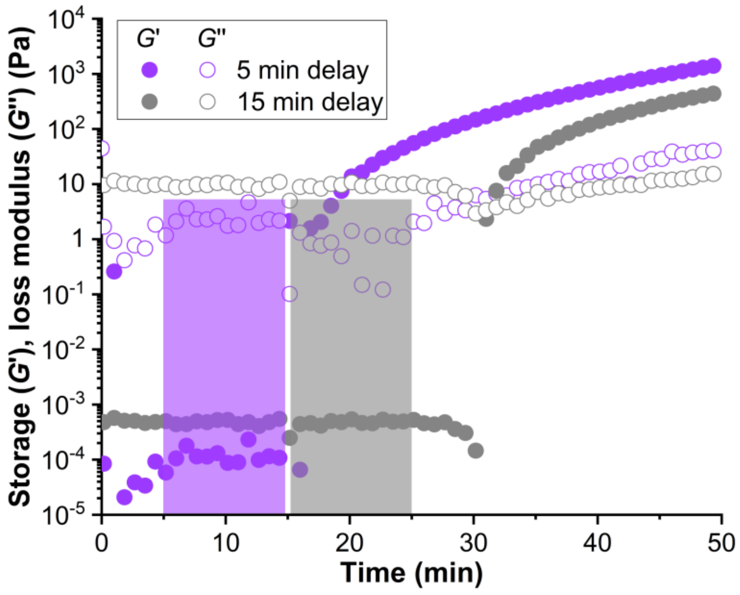

Figure S17. Time-dependent oscillatory rheological test showing light-triggered gelation of P(BLG-*co*-HCys), with gel points consistently appearing approximately 14 min after the onset of irradiation, regardless of whether the light was applied after 5 or 15 min. Illumination periods are depicted with violet or grey rectangle.

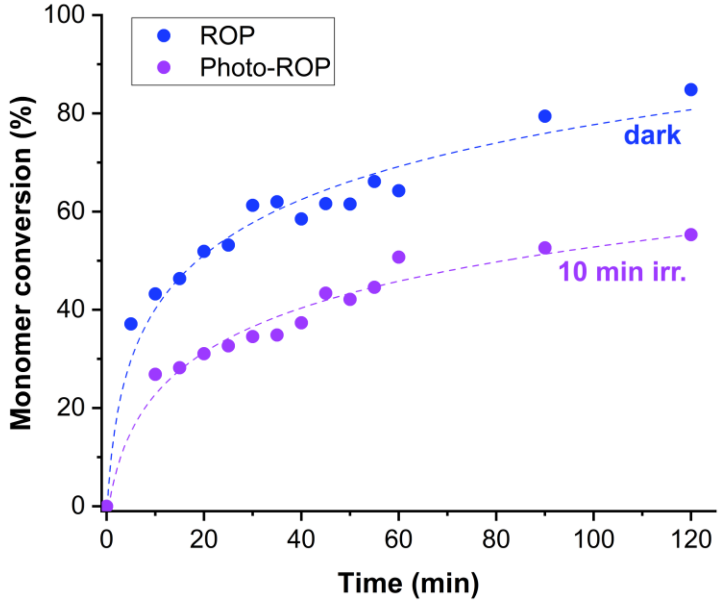

Figure S18. Monomer conversion as a function of time for *co*-ROP of BLG NCA and HCys NCA: Photo-ROP with PB-DBA under 10 min of irradiation, and with DBA without irradiation.

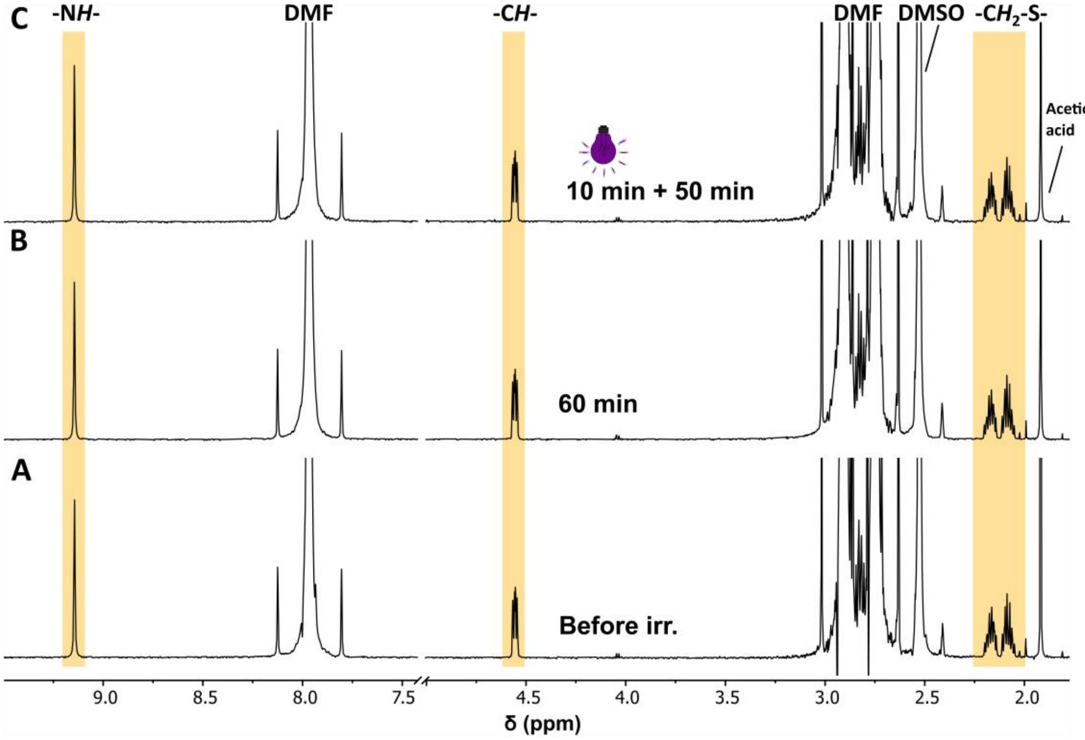

Figure S19. ^{1}H NMR spectra of HCys NCA in anhydrous DMF (0.08 M) with TFA (6.25 % relative to HCys NCA): **A**) before irradiation; **B**) after 60 min in the absence of light; **C)** after 10 min of irradiation (365 nm, 90 mW·cm^{-2}), followed by 50 min in the absence of light. The spectra were recorded in DMSO-d_6 with added TFA.

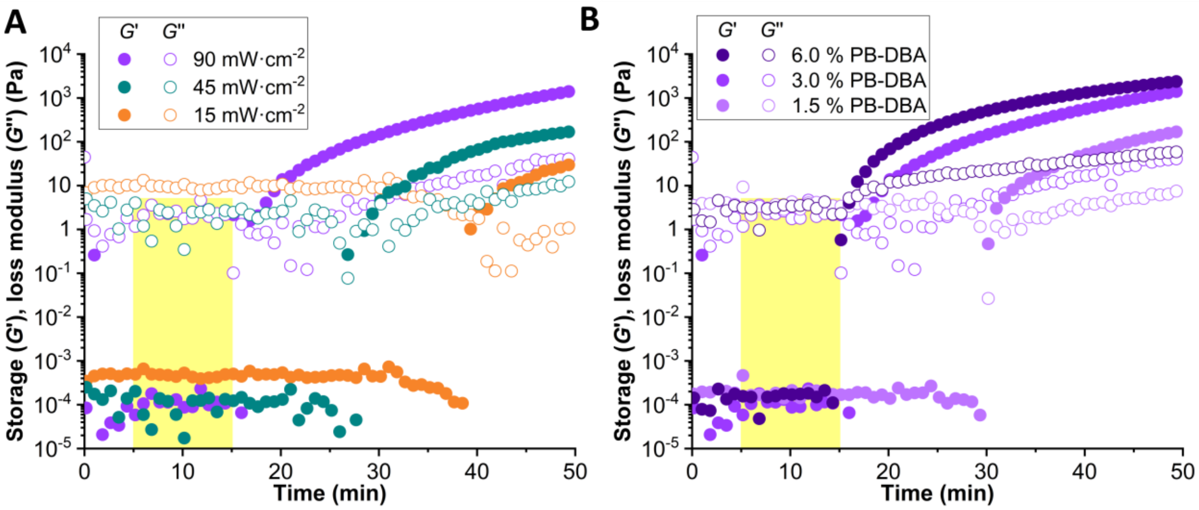

Figure S20. Time-dependent oscillatory test results for the photoinduced ring-opening copolymerization of BLG NCA and HCys NCA: **A**) at different light intensities; **B**) at different PB-DBA initial concentrations.

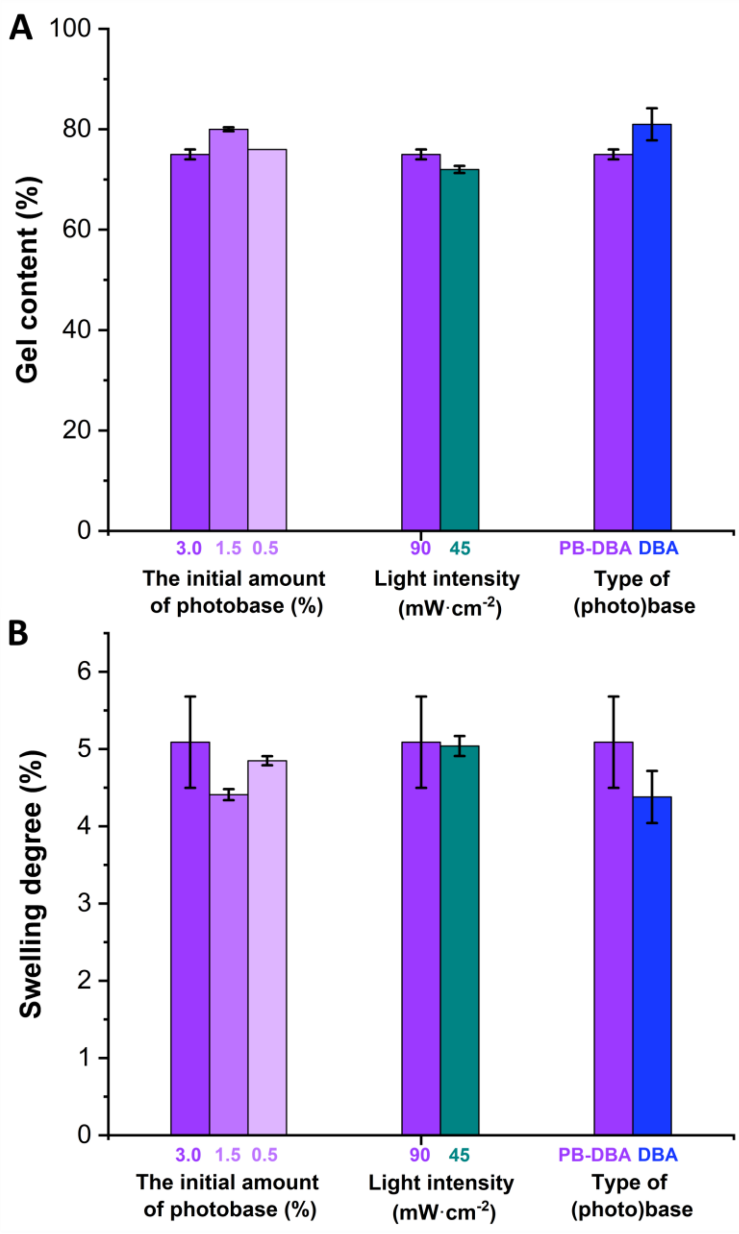

Figure S21. A) Gel content and **B**) swelling degree values of P(BLG-*co*-HCys) gels at different initial concentrations of photobase, at different light intensities and type of (photo)base.

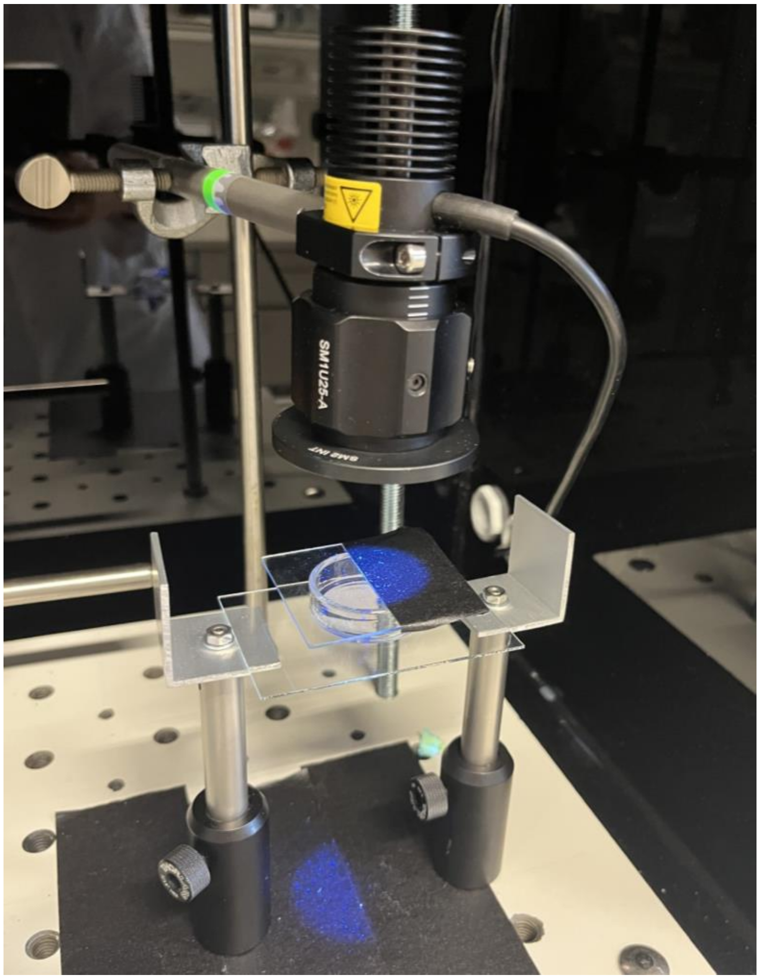

Figure S22. Set-up of a photomask experiment, showing irradiation of a sample from above. The sample was covered with a glass slide, which was shielded by a UV-impermeable plate of optional shape.

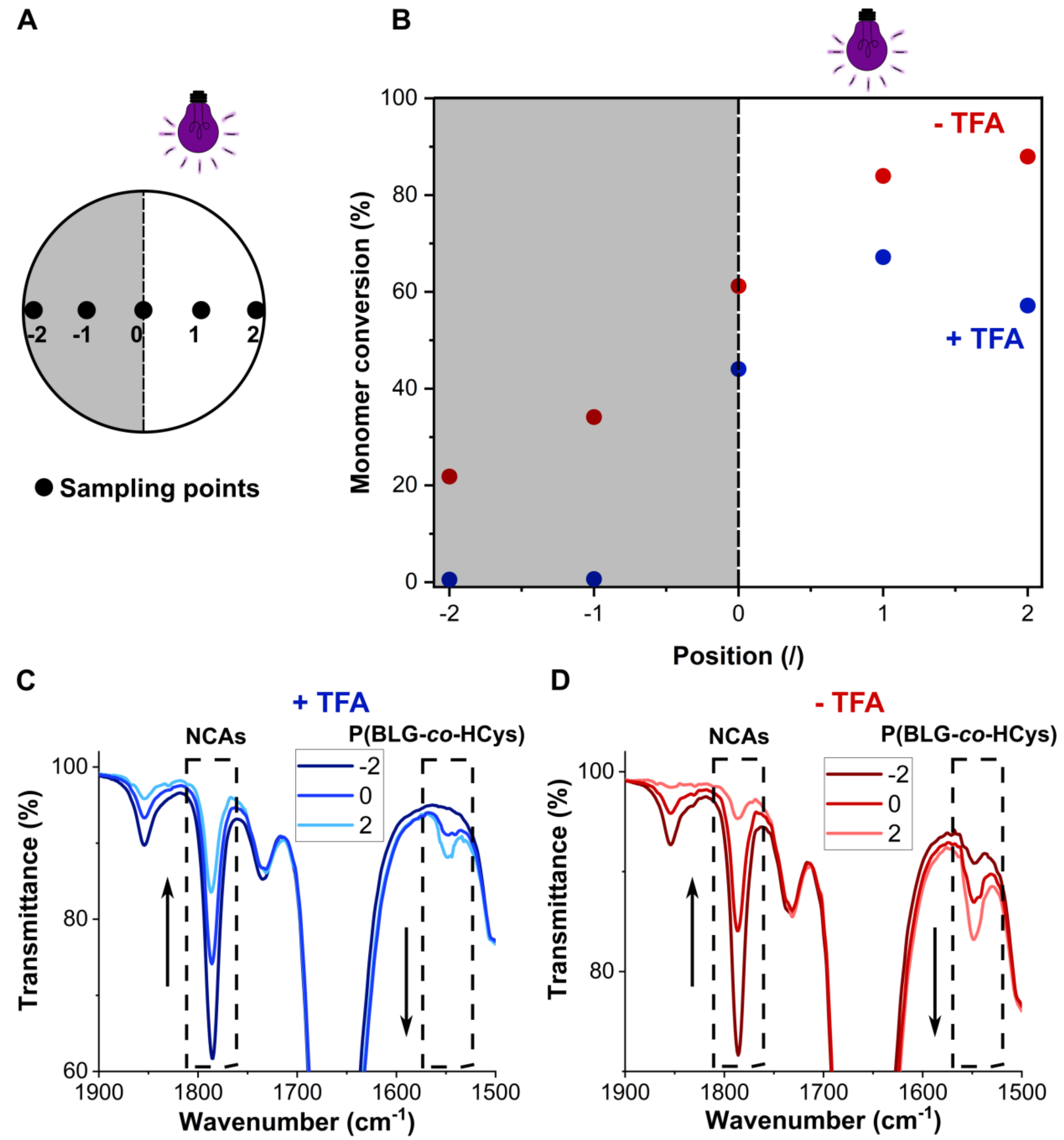

Figure S23. A) Schematic representation of the half-irradiated sample in the photomask experiment, showing sampling points for FTIR being approximately 7 mm apart. Positions -2 and -1 indicate the non-irradiated side, position 0 is the centre, and positions 1 and 2 are the irradiated parts of the reaction mixture; **B**) Monomer conversion as a function of position for photo-ROP after 12 min irradiation, **with (+)** and **without (-)** TFA; **C**) FTIR spectra recorded at different positions of the reaction mixture (**with (+) TFA**) after 12 min of irradiation in the photomask experiment; **D**) FTIR spectra recorded at different positions of the reaction mixture (**without (-) TFA**) after 12 min of irradiation in the photomask experiment.

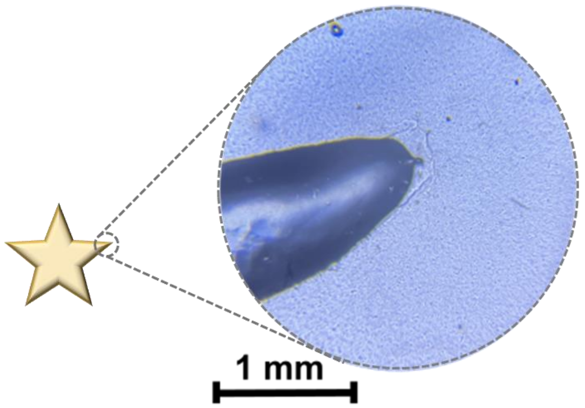

Figure S24: Optical microscopy image of the tip of an arm of the star-shaped gel at 40× magnification.

References

[1] Š. Gradišar, E. Žagar, D. Pahovnik, “Ring-Opening Polymerization of *N*-Carboxyanhydrides Initiated by a Hydroxyl Group” *ACS Macro Lett.* **2017**, *6*, 637−640.

[2] P. Utroša, E. Žagar, D. Pahovnik, “Insight into the cross-linking of synthetic polypeptide gels prepared by ring-opening polymerization using l-cystine *N*-carboxyanhydride” *Eur. Polym. J.* **2024**, *204*, 112707.

[3] S. P. Rannard, N. J. Davis, “The Selective Reaction of Primary Amines with Carbonyl Imidazole Containing Compounds: Selective Amide and Carbamate Synthesis” *Org. Lett.* **2000**, *2*, 2117–2120.

[4] J. C. Foster, A. W. Cook, N. T. Monk, B. H. Jones, L. N. Appelhans, E. M. Redline, S. C. Leguizamon, “Continuous Additive Manufacturing using Olefin Metathesis” *Adv. Sci.* **2022**, *9*, 2200770.
